# Immunization using ApoB-100 peptide–linked nanoparticles reduces atherosclerosis

**DOI:** 10.1172/jci.insight.149741

**Published:** 2022-06-08

**Authors:** Kuang-Yuh Chyu, Xiaoning Zhao, Jianchang Zhou, Paul C. Dimayuga, Nicole W.M. Lio, Bojan Cercek, Noah T. Trac, Eun Ji Chung, Prediman K. Shah

**Affiliations:** 1 Oppenheimer Atherosclerosis Research Center, Department of Cardiology, Smidt Heart Institute, Cedars-Sinai Medical Center, Los Angeles, California, USA.; 2 Department of Biomedical Engineering, University of Southern California, Los Angeles, California, USA.

**Keywords:** Cardiology, Vaccines, Adaptive immunity, Atherosclerosis, T cells

## Abstract

Active immunization with the apolipoprotein B-100 (ApoB-100) peptide P210 reduces experimental atherosclerosis. To advance this immunization strategy to future clinical testing, we explored the possibility of delivering P210 as an antigen using nanoparticles, given this approach has been used clinically. We first characterized the responses of T cells to P210 using PBMCs from patients with atherosclerotic cardiovascular disease (ASCVD). We then investigated the use of P210 in self-assembling peptide amphiphile micelles (P210-PAMs) as a vaccine formulation to reduce atherosclerosis in *B6.129P2-Apoe^tm1Unc^/J* (*ApoE^–/–^*) mice and P210’s potential mechanisms of action. We also generated and characterized a humanized mouse model with chimeric HLA-A*02:01/Kb in *ApoE^–/–^* background to test the efficacy of P210-PAM immunization as a bridge to future clinical testing. P210 provoked T cell activation and memory response in PBMCs of patients with ASCVD. Dendritic cell uptake of P210-PAM and its costaining with MHC-I molecules supported its use as a vaccine formulation. In *ApoE^–/–^* mice, immunization with P210-PAMs dampened P210-specific CD4^+^ T cell proliferative response and CD8^+^ T cell cytolytic response, modulated macrophage phenotype, and significantly reduced aortic atherosclerosis. Potential clinical relevance of P210-PAM immunization was demonstrated by reduced atherosclerosis in the humanized *ApoE^–/–^* mouse model. Our data support experimental and translational use of P210-PAM as a potential vaccine candidate against human ASCVD.

## Graphical abstract

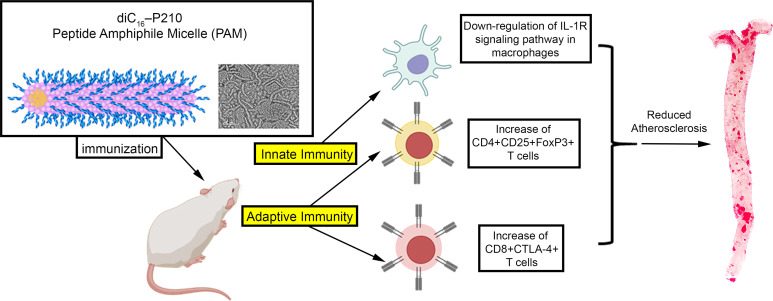

## Introduction

Adaptive immune response against self-antigens such as LDL, apolipoprotein B-100 (ApoB-100), or certain ApoB-100–related peptide epitopes is a hallmark of experimental and human atherosclerosis (1–3). Among the ApoB-100 peptides, P210 is the subject of several investigations to develop antigen-specific immune modulation (4–7). We have previously demonstrated P210-specific CD8^+^ T cells in hypercholesterolemic mice can be detected by peptide-loaded synthetic soluble MHC-I pentamers. These P210-specific CD8^+^ T cells increased in response to atherogenic diet, correlated with the extent of atherosclerosis, and localized to atherosclerotic plaques (8). Additionally, P210 fragments and P210-specific antibodies have been detected in plaques and circulation of patients with atherosclerotic cardiovascular disease (ASCVD), suggesting the involvement of P210 in human atherosclerotic disease (9, 10).

An outcome of various experimental strategies of P210 immune modulation is alteration of T cell responses to P210, suggesting that the peptide or derivatives thereof are self-antigens that provoke immune responses involved in atherosclerosis (5, 11). We have demonstrated that P210, when used in an active immunization strategy, elicits CD8^+^ T cell response to reduce atherosclerosis (4), potentially by shifting the immune-dominant epitope (8). These experimental observations implicate immune response to P210 in atherogenesis and suggest that modification of the intrinsic immune response to P210 could reduce human atherosclerosis.

In preclinical studies, immunogenic peptides are often conjugated as haptens to carrier molecules along with an adjuvant such as mineral salt to provoke an immune response to establish vaccine efficacy. Traditional aluminum salt–based vaccines are known to induce weak cell-mediated immune responses, limiting their clinical application and choice of antigens (12, 13). The evidence from work with P210 immunization in animal models shows the involvement of various cellular immune responses such as regulatory T cell or CD8^+^ T cell responses (4, 5, 11, 14). We therefore surmised that an approach targeting immune regulation of the response to P210 would be beneficial in atherosclerosis. One effective way to deliver antigens that provoke a regulatory response is to use the nanoparticle platform (15).

Mechanistically, nanoparticles have favorable physicochemical properties that provide size-preferential lymphatic transport, relatively long injection site retention and circulating time for contact with dendritic cells acting as adjuvants in subunit vaccines, and the induction of autoimmunity-specific regulatory immune responses (16). A variety of nanoparticle platforms have been tested to target inflammation and to modulate immune function in atherosclerosis with wide potential in humans (17–21). More importantly, nanoparticle-based vaccines are already in clinical use to prevent COVID-19 infection and being tested in a clinical trial to treat autoimmune disease, such as celiac disease (22).

In this study, we first assessed the clinical relevance of P210 as a self-antigen in ASCVD by demonstrating that P210 provokes T cell responses in patients with acute coronary syndrome. We then utilized the peptide amphiphile nanoparticle platform in which the P210 is chemically conjugated to hydrophobic tails, facilitating subsequent self-assembly into well-defined peptide amphiphile micelles (PAMs) (23–25). PAMs were used as nanocarriers because they are composed of biocompatible lipids and peptides and are chemically versatile, allowing the incorporation of multiple modalities, such as fluorescence and immunogenicity, into a single particle. We tested if the PAM nanoparticle platform can function as a vaccine formulation to reduce atherosclerosis in hypercholesterolemic *ApoE^–/–^* mice and explored its potential mechanisms of achieving such an effect. As a bridge to potential human testing, we developed a chimeric HLA-A*02:01/Kb ApoE^–/–^ humanized mouse model (*A2Kb-Tg ApoE^–/–^* mice) to test the efficacy of P210-PAM. The focus on class I HLA-A*02:01 is supported by our previous report demonstrating the importance of the MHC-I/CD8^+^ T cell pathway in both the intrinsic immune response to P210 as well as potential immune-modulating therapy (4, 8). Herein, we report the effects of P210-PAM immunization on immune responses in atherosclerosis and tested the translational application of the P210-PAM formulation as a potential human vaccine using *A2Kb-Tg ApoE^–/–^* mice.

## Results

### Intrinsic T cell response to ApoB-100 peptide P210 in patients with acute coronary syndrome.

We previously demonstrated that immune modulation of T cells reactive with the ApoB-100 peptide P210 in *ApoE^–/–^* mice reduces atherosclerosis (4). To evaluate if self-reactive T cell response to P210 is present in humans, we investigated the intrinsic T cell response to P210 in humans by testing peripheral blood mononuclear cells (PBMCs) from patients with acute coronary syndrome (ACS) and self-reported healthy volunteers as controls. Patients with ACS were selected for this exploratory study because their unequivocal ASCVD. Patient characteristics are in Table 1.

**Figure 1:**
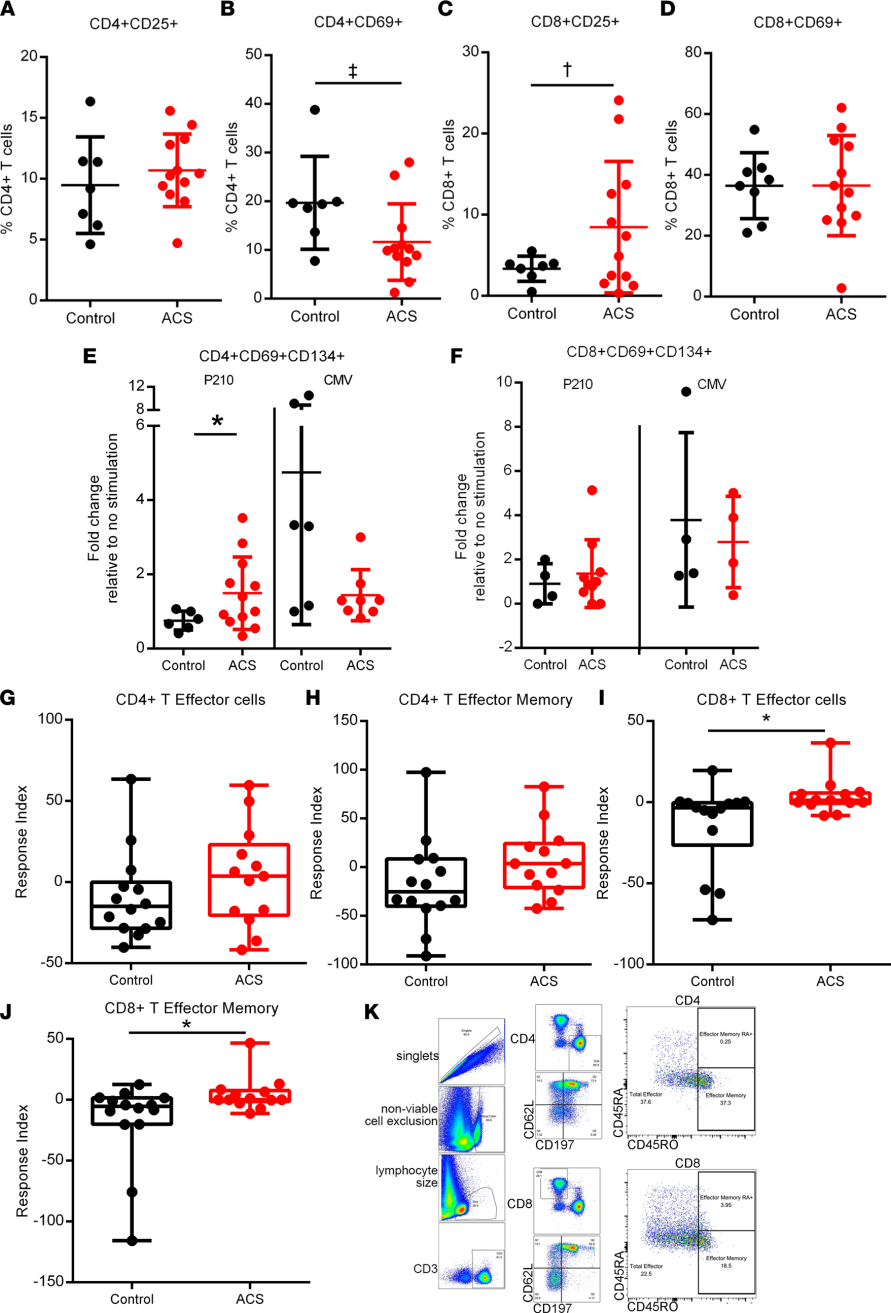
Intrinsic T cell response to P210 peptide in human PBMCs. Human PBMCs from control patients and acute coronary syndrome (ACS) patients were cultured for 16 hours for the AIM assay with no stimulation or stimulated with P210 peptide or CMV pooled peptides. (**A**–**D**) Activation state of PBMCs without peptide stimulation. (**E** and **F**) AIM^+^ cells in response to P210 or CMV peptide pool. (**G**–**J**) PBMCs were stimulated with P210 peptide for 72 hours, and cells were stained for T effector and memory markers. The box plots depict the minimum and maximum values (whiskers), the upper and lower quartiles, and the median. The length of the box represents the interquartile range. (**K**) Gating scheme for T effector and memory cell analysis. Mann-Whitney *U* test except for **C** and **E**, 2-tailed *t* test. ^‡^*P* = 0.07; ^†^*P* = 0.05; *P < 0.05. (**A**–**F**) Control *n* = 7–8, ACS *n* = 12; some samples/treatments did not have detectable AIM^+^ cells so ratio could not be determined. (**G**–**J**) Control *n* = 14, ACS *n* = 13.

In order to determine if P210 is capable of activating T cells as an antigen, we conducted an activation-induced marker (AIM) assay (26–28). At baseline, there were fewer CD4^+^CD69^+^ T cells and greater CD8^+^CD25^+^ T cells in PBMCs from patients with ACS compared with controls, whereas no difference in CD4^+^CD25^+^ and CD8^+^CD69^+^ T cells between the 2 groups was noted (Figure 1, A–D; *P* = 0.07 for Figure 1B; *P* = 0.05 for Figure 1C). AIM assay demonstrated a mean 1.5-fold increase in CD4^+^CD69^+^CD134^+^ T cells after P210 stimulation in patients with ACS compared with controls, while no such increase was observed in CD8^+^CD69^+^CD134^+^ T cells (Figure 1, E and F). CMV pooled peptide (right panel in Figure 1, E and F) or cell stimulation cocktail (PMA/ionomycin, Supplemental Figure 1, A–F; supplemental material available online with this article; https://doi.org/10.1172/jci.insight.149741DS1) as positive controls validated the AIM assay. We did not observe differences in CD25^+^CD134^+^, CD69^+^CD154^+^, or CD134^+^CD137^+^ in either CD4^+^ or CD8^+^ T cells (Supplemental Figure 1, G–L). Although a cutoff of 2-fold increase may be appropriate in studying T cell activation to exogenous antigens (infectious or vaccine antigens), T cell responses to intrinsic self-antigens are not expected to be as robust, since the immune-inflammatory response to self-antigens in autoimmune diseases tends to be chronic and low-grade.

A hallmark feature of adaptive immune response is the recall response of antigen-experienced T cells to antigen reexposure. Given patients with ACS have definite atherosclerosis, we tested if their T cells would generate such a recall response to P210 restimulation. CD4^+^ T effector cell response to P210 was not significantly different in the ACS PBMCs compared to controls (Figure 1, G and H). However, there was a significant increase in CD8^+^ T effector (Figure 1I), and CD8^+^ T effector memory (Figure 1J; gating strategy for T cells in Figure 1K) response in ACS PBMCs compared with controls, which supports the existence of antigen-experienced, P210-specific T cells in humans with atherosclerosis.

### Characteristics of P210 peptide.

The T cell response observed in PBMCs from patients with ACS suggested that P210 may be a self-peptide that provokes a self-reactive immune response. It remains unknown how ApoB-100 peptides become immunogenic, but the presence of antibodies against ApoB-100 peptides in patients with ASCVD suggests the potential of antigen-presenting cells (APCs) to present peptides derived from LDL particles that have undergone oxidation and subsequent breakdown (9, 29–31). Indeed, various ApoB-100 peptide fragments, including P210, have been detected in atherosclerotic plaques by mass spectrometry (10). However, it remains unknown how ApoB-100 peptides, specifically P210, are able to enter dendritic cells (DCs) to function as intrinsic self-antigens.

**Figure 2:**
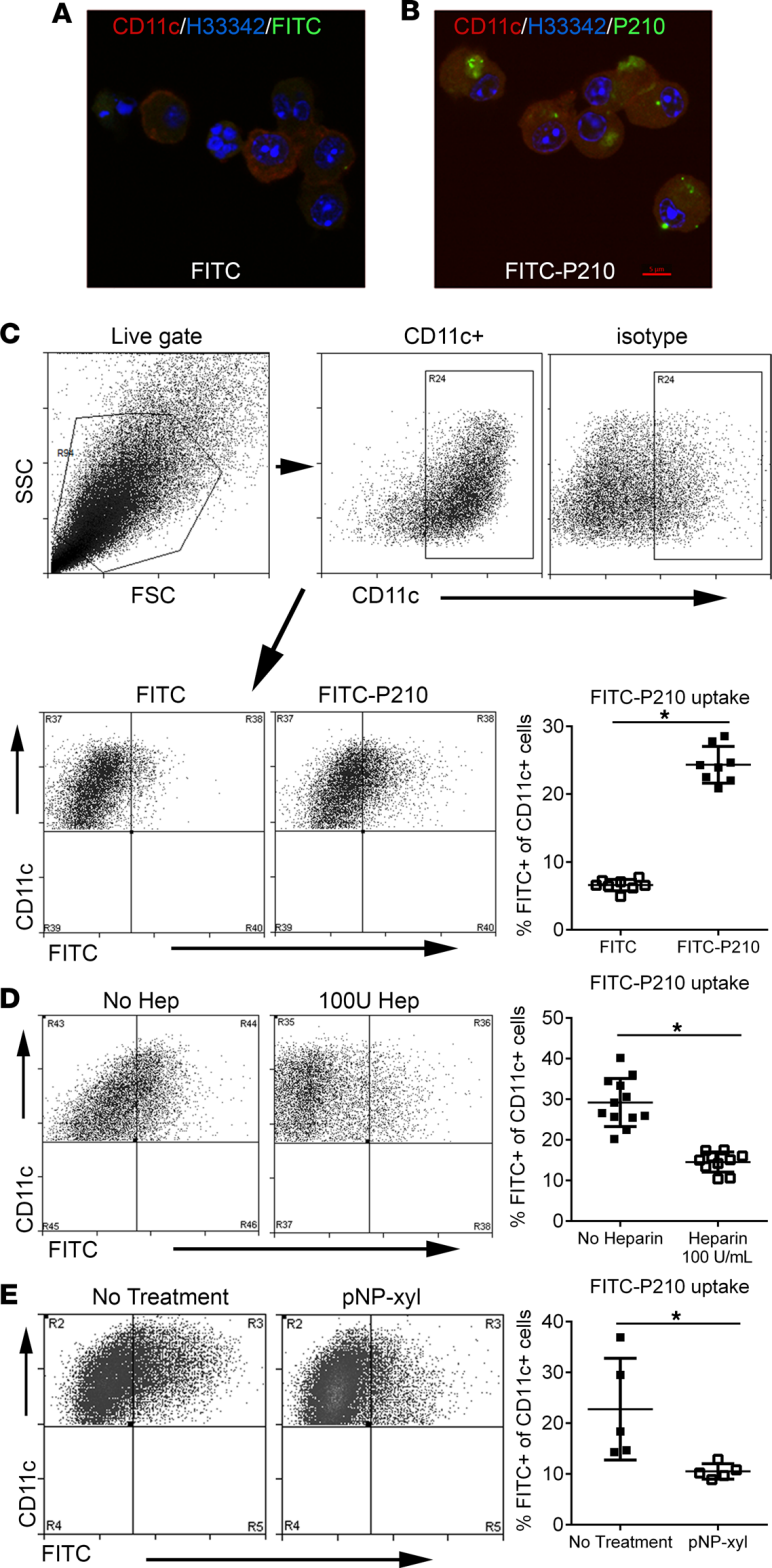
P210-FITC uptake by mouse bone marrow–derived DCs. Confocal microscopy of bone marrow–derived DCs (BMDCs) incubated with (**A**) FITC only or (**B**) P210-FITC. Same magnification in **A** and **B** and red bar = 5 μm in **B**. (**C**) FITC internalization was quantified using flow cytometry of CD11c-stained cells. Cells were size gated and then gated on CD11c (**C**, top panel). CD11c^+^ cells were then analyzed on CD11c/FITC quadrants and the results plotted on a scatter graph indicating the mean percentage of FITC^+^ cells on the CD11c^+^ gate (**C**, bottom panel; *n* = 8 each). (**D**) Heparin binds P210-FITC (no heparin *n* = 12; 100 U heparin *n* = 10). (**E**) Proteoglycan inhibitor p-Nitrophenyl β-D-xylopyranoside (pNP-xyl) blocks proteoglycan-mediated uptake of P210-FITC (*n* = 5 each). *P < 0.05, 2-tailed *t* test.

P210 is a cationic peptide fragment that is within the proteoglycan-binding domain of ApoB-100 that has the properties of a cell-penetrating peptide (CPP). Cationic CPPs are rich in positively charged Arg and Lys residues, which enable interaction with negatively charged cell surface proteoglycans (32, 33). Given the Lys-rich sequence of P210 (**K**TT**K**QSFDLSV**K**AQY**KK**N**K**H) and a calculated isoelectric point of 10.85, we investigated if P210 could enter mouse bone marrow–derived DCs through the proteoglycan pathway. We first used confocal microscopy to visualize the uptake of FITC-conjugated P210 peptides (P210-FITC) into CD11c^+^ DCs, and flow cytometric analysis confirmed significantly increased uptake of P210-FITC (Figure 2, A–C). The proteoglycan-binding capacity of P210 was assessed by using heparan to block DC uptake (34), and P210-FITC entry into DCs was significantly reduced by 100 U/mL of heparan (Figure 2D). To confirm that the cellular uptake of P210 is mediated by cell surface proteoglycan binding, DCs were treated with p-nitrophenyl-β-D-xylopyranoside (pNP-xyl), a competitive inhibitor of heparan sulfate chain addition, preventing the synthesis of functional cell surface heparan sulfate proteoglycans (35). Treatment of DCs with pNP-xyl significantly reduced P210-FITC entry (Figure 2E), supporting the notion that P210 uptake by DCs is mediated in part through cell surface proteoglycan binding. The results demonstrate that P210 has properties of a CPP that enables its entry into APCs such as DCs and potentially presented to T cells as a self-peptide.

### Immune modulation and biodistribution of P210 nanoparticles.

**Figure 3:**
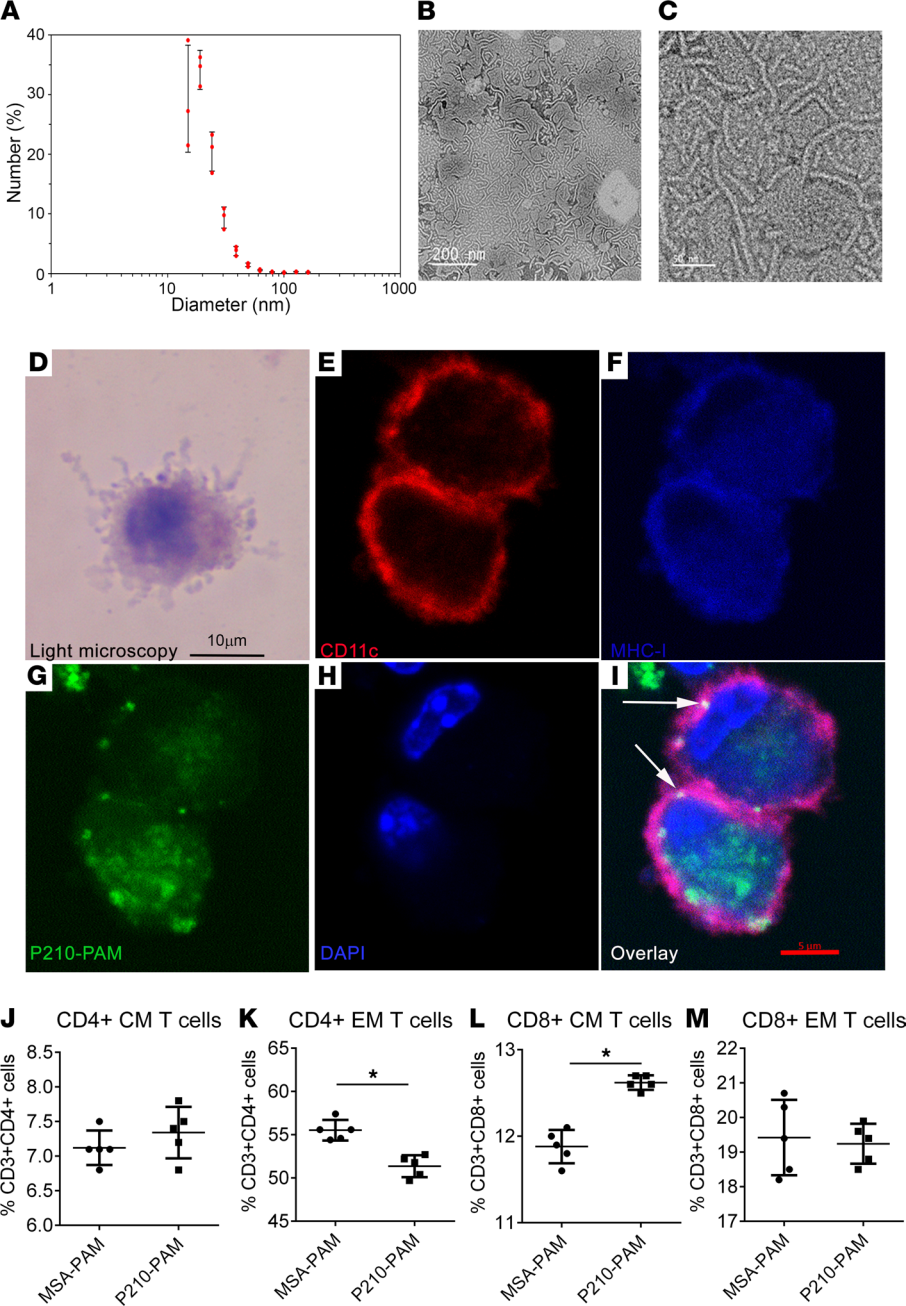
P210-PAM nanoparticles. (**A**) The majority of P210-PAMs are between 15 and 25 nm (*n* = 3). Transmission electron microscopy of P210-PAM at low (**B**) and high (**C**) magnification. Scale bars: 200 nm (**B**), 50 nm (**C**). (**D**) Light microscopy of Giemsa-stained mouse BMDCs. Fixed BMDCs stained with (**E**) CD11c PE, (**F**) MHC-I APC, (**G**) FITC-P210-PAM, and (**H**) DAPI. (**I**) Color overlay and arrows indicating costaining. The last lysine of the P210 peptide was FITC labeled prior to PAM assembly. The experiment was replicated twice with similar results. Scale bars: 10 μm (**D**–**F**), 5 μm (**G**–**I**).(**J**) CD4^+^ central memory (CM) T cells, (**K**) CD4^+^ effector memory (EM) T cells, (**L**) CD8^+^ CM T cells, and (**M**) CD8^+^ EM T cells from spleens of 25-week-old *ApoE^–/–^* mice fed a high-cholesterol diet for 16 weeks. Splenocytes were collected after 48 hours’ treatment with 20 μg/mL MSA-PAM or P210-PAM. *n* = 5 each, *P < 0.05 by 2-tailed *t* test.

To enable efficient antigen delivery by protecting peptides from protease degradation and clearance and providing a scaffold for increased epitope density, P210 was incorporated into PAMs through covalent conjugation of the peptide to 1*′*-3*′*-dihexadecyl *N*-succinyl-_L_-glutamate (diC_16_) hydrophobic moieties. Hydrophobic interaction induced self-assembly of the diC_16_-P210 monomers into cylindrical micelles with an average diameter of 21.6 ± 1.1 nm, a polydispersity index of 0.152 ± 0.001, and a zeta potential of 2.7 ± 0.8 mV (Figure 3, A–C; Supplemental Figure 2; and Supplemental Figure 3).

First, we tested whether P210-PAM enters DCs and if P210 (or its fragment) can be costained with MHC-I by conducting confocal experiments using FITC-labeled P210-PAM. MHC-I was chosen as the pathway to visualize given prior data indicating the involvement of the MHC-I/CD8^+^ T cell pathway in P210 immunization, consistent with the reported characterization of CPPs to be cross-presented to MHC-I (34, 36). Confocal microscopy demonstrated costaining of FITC-labeled P210 with MHC-I molecule on the surface of mouse DCs (Figure 3, D–I).

P210-PAM was then tested for reactivity with T cells of *ApoE^–/–^* mice, and mouse serum albumin PAMs (MSA-PAMs) were used as a control. There were a significant reduction in CD4^+^ effector memory T cells and an increase in CD8^+^ central memory T cells treated with P210-PAM when compared with MSA-PAM–treated splenocytes of *ApoE^–/–^* mice fed a high-cholesterol diet for 16 weeks (Figure 3, K and L). Although central memory CD4^+^ T cells and effector memory CD8^+^ T cells remained unchanged (Figure 3, J and M), the results suggest that P210-PAM provokes a memory T cell response in naive hypercholesterolemic *ApoE^–/–^* mice.

**Figure 4:**
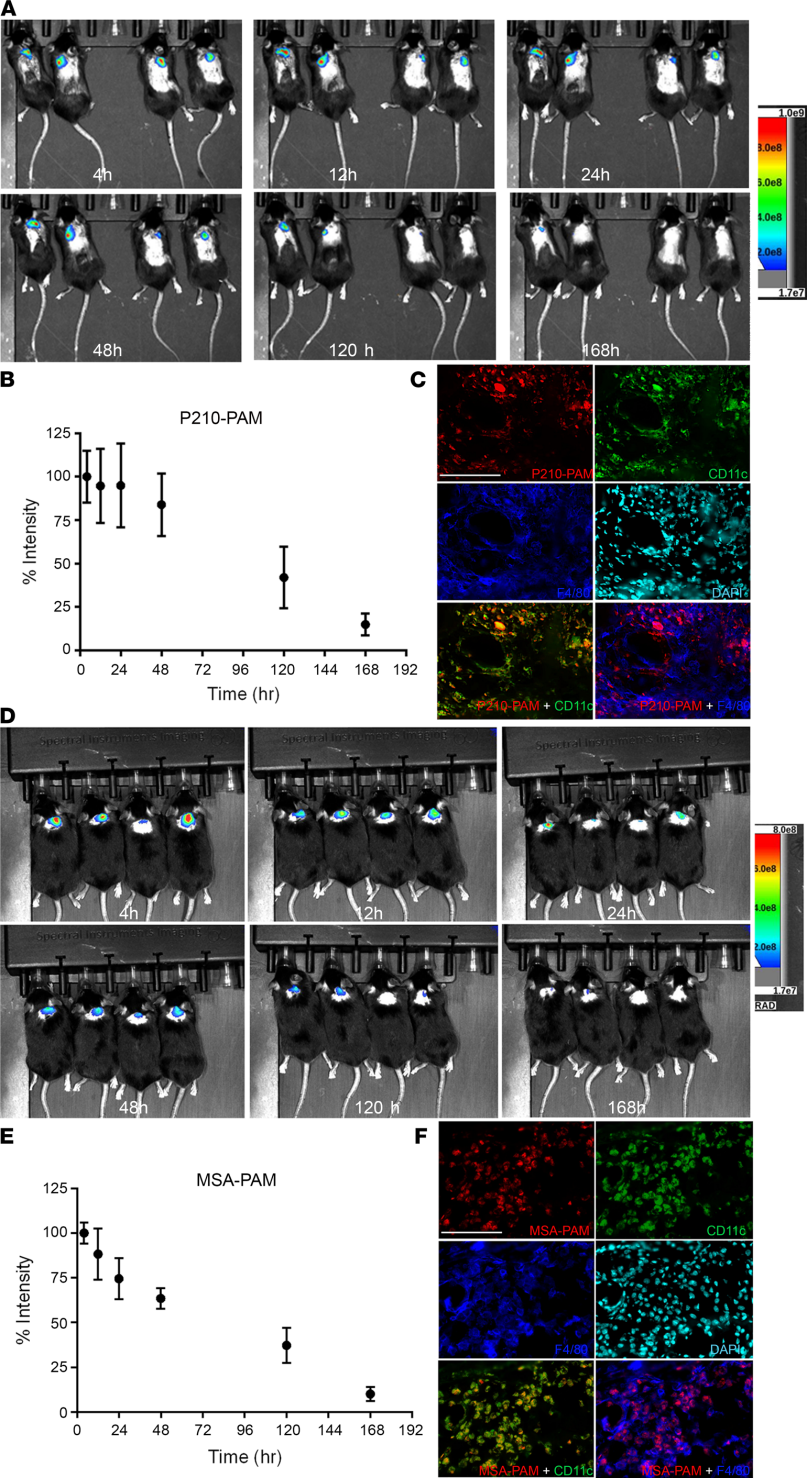
PAM imaging and retention in vivo. In vivo imaging of P210-PAM (**A**–**C**) or MSA-PAM (**D**–**F**) retention at the injection site of *C57BL/6J* mice over 168 hours. Percentage of signal intensity relative to time 0 (immediately after injection) of P210-PAM (**B**) or MSA-PAM (**E**). *n* = 4 each. Colocalization of fluorescently labeled P210-PAM (**C**) or MSA-PAM (**F**) with F4/80^+^ macrophages and CD11c^+^ DCs at the injection site at 48 hours.

Effective immunization depends not only on the immunogenicity of antigens but also on their retention at the injection site (37). We hence characterized the biodistribution kinetics of fluorescently labeled P210-PAM injected subcutaneously into wild-type mice and imaged over a period of 7 days, showing 80%, 30%, and 15% retention in the injection site at 2, 5, and 7 days, respectively, with a calculated clearance half-life of 79.7 ± 29.2 hours (Figure 4, A and B). Immunofluorescence staining of the injection site showed colocalization of P210-PAM with F4/80^+^ macrophages and CD11c^+^ DCs (Figure 4C). MSA-PAM had percentage retention of 67%, 37%, and 11% at 2, 5, and 7 days, respectively, and a clearance half-life of 72.7 ± 29.2 hours (Figure 4, D–F).

### Nanoparticle-based immune modulation of T cell responses to P210-PAM immunization.

**Figure 5:**
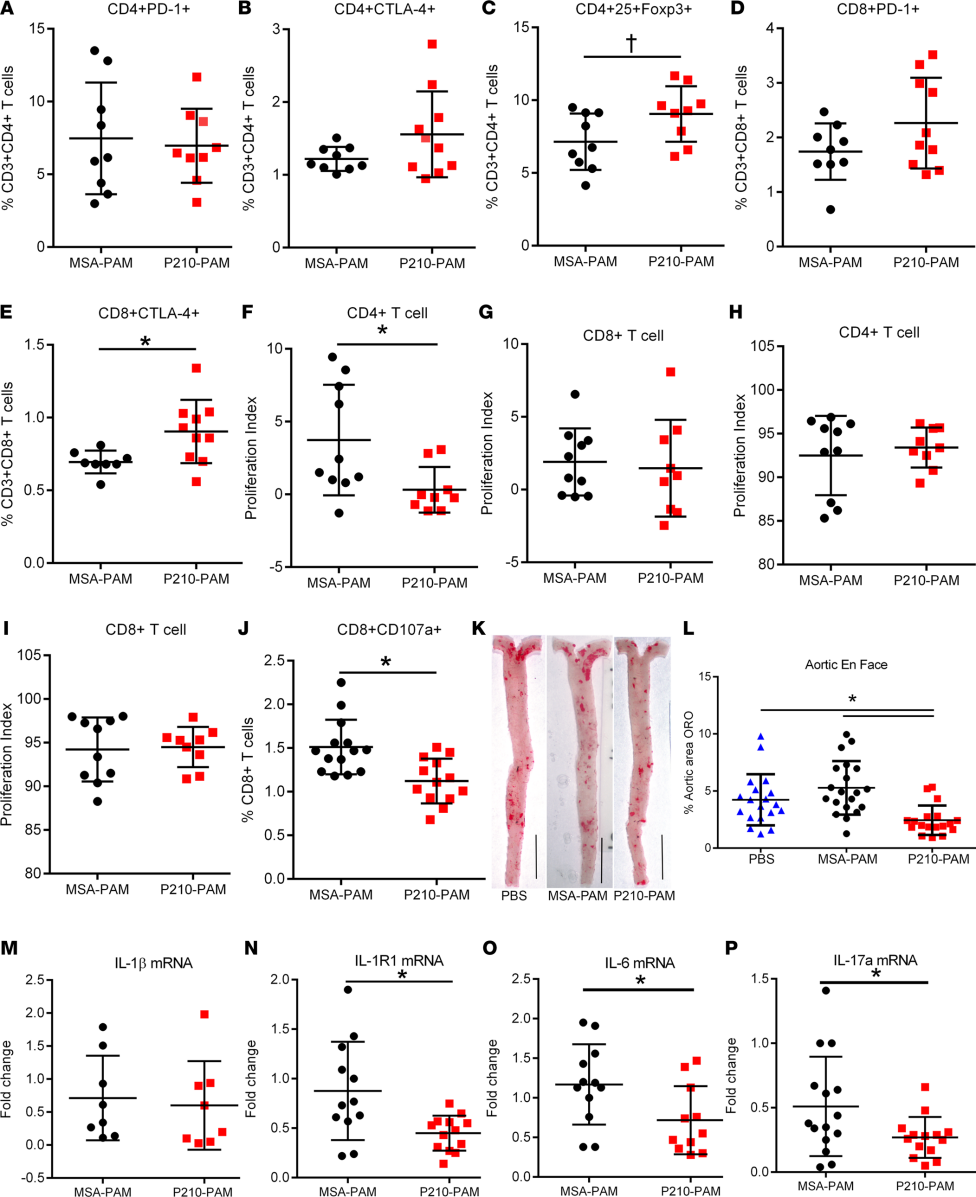
P210-PAM immunization in *ApoE^–/–^* mice. (**A**–**C**) Immune regulatory profile of CD4^+^ and (**D** and **E**) CD8^+^ T cells in splenocytes of immunized mice 1 week after second booster. (**F** and **G**) Splenic T cell proliferation of immunized mice in response to P210 peptide or (**H** and **I**) Concanavalin A (Con A) stimulation assessed by BrdU staining. (**J**) CD107a to assess CD8^+^ T cell cytolytic activity in splenocytes of immunized mice. (**K**) Representative photographs of aortic en face staining with oil red O at 25 weeks of age. (**L**) Atherosclerosis measured as percentage of whole aorta stained by oil red O. Splenic mRNA expression of (**M**) IL-1β, (**N**) IL-1R1, (**O**) IL-6, and (**P**) IL-17a. Number of mice used in each group is represented by the number of dots in individual figure. *P < 0.05; ^†^*P* = 0.05, 2-tailed *t* test except for **L**, 1-way ANOVA with Holm-Šidák multiple comparisons test.

The effect of P210-PAM immunization on immune regulation was then tested in *ApoE^–/–^* mice, using the MSA-PAM as a control. Immunized male *ApoE^–/–^* mice euthanized 1 week after the second booster injection showed no differences in splenic CD4^+^ programmed cell death 1–positive (PD-1^+^) and CD4^+^ cytotoxic T lymphocyte–associated protein 4–positive (CTLA-4^+^) T cells between P210-PAM– and MSA-PAM–immunized mice (Figure 5, A and B). CD4^+^CD25^+^FoxP3^+^ regulatory T cells were increased in P210-PAM–immunized mice compared with those immunized with MSA-PAM (Figure 5C, P = 0.05). There were no differences in CD8^+^PD-1^+^ T cell numbers (Figure 5D), but CD8^+^CTLA-4^+^ T cells were significantly increased in P210-PAM–immunized mice compared with MSA-PAM–immunized mice (Figure 5E). CD4^+^ T cells from P210-PAM–immunized mice had significantly reduced proliferative response to P210 stimulation compared with CD4^+^ T cells from MSA-PAM–immunized mice (Figure 5F), but this was not observed in CD8^+^ T cells (Figure 5G). CD4^+^ T cells (Figure 5H) and CD8^+^ T cells (Figure 5I) responded to Con A stimulation similarly between the 2 groups, suggesting specificity of the regulation of T cell response. Even though P210-PAM immunization had no effect on CD8^+^ T cell proliferation, there was reduced cytolytic function of CD8^+^ T cells in response to P210 stimulation in P210-PAM–immunized mice compared with MSA-PAM–immunized mice as determined by CD107a staining (Figure 5J). Thus, P210-PAM provoked antigen-specific effects as well as regulation of CD4^+^ T cells’ proliferation and CD8^+^ T cells’ cytolytic function. No differences were observed in DC phenotypes (Supplemental Figure 4).

### P210-PAM immunization reduced atherosclerosis in ApoE^–/–^ mice.

To test the effect of P210-PAM immunization on atherosclerosis, *ApoE^–/–^* mice were subjected to the same immunization schedule described above and then fed a high-cholesterol diet from 13 weeks of age until euthanasia at 25 weeks of age. En face oil red O staining of the aorta (Figure 5K) showed significantly reduced aortic atherosclerosis in P210-PAM–immunized mice compared with PBS- and MSA-PAM–immunized mice (Figure 5L). The mean circulating levels of total cholesterol or LDL-C in P210-PAM–immunized mice were lower than those in MSA-PAM–immunized mice but similar to the mean levels in PBS mice, whereas there was no difference in circulating level of HDL-C among the 3 groups (Supplemental Figure 5, A–C). There was no difference in IgM or IgG level against P210 among groups, but the P210-PAM–immunized group had reduced IgG1 and IgG2b against P210 (Supplemental Figure 5, D–G). No differences were observed in the aortic sinus plaque size, lipid stain, and macrophage content (Supplemental Figure 6, A–I).

### P210-PAM immunization reduces IL-1R1 expression and modulates macrophage phenotype.

**Figure 6:**
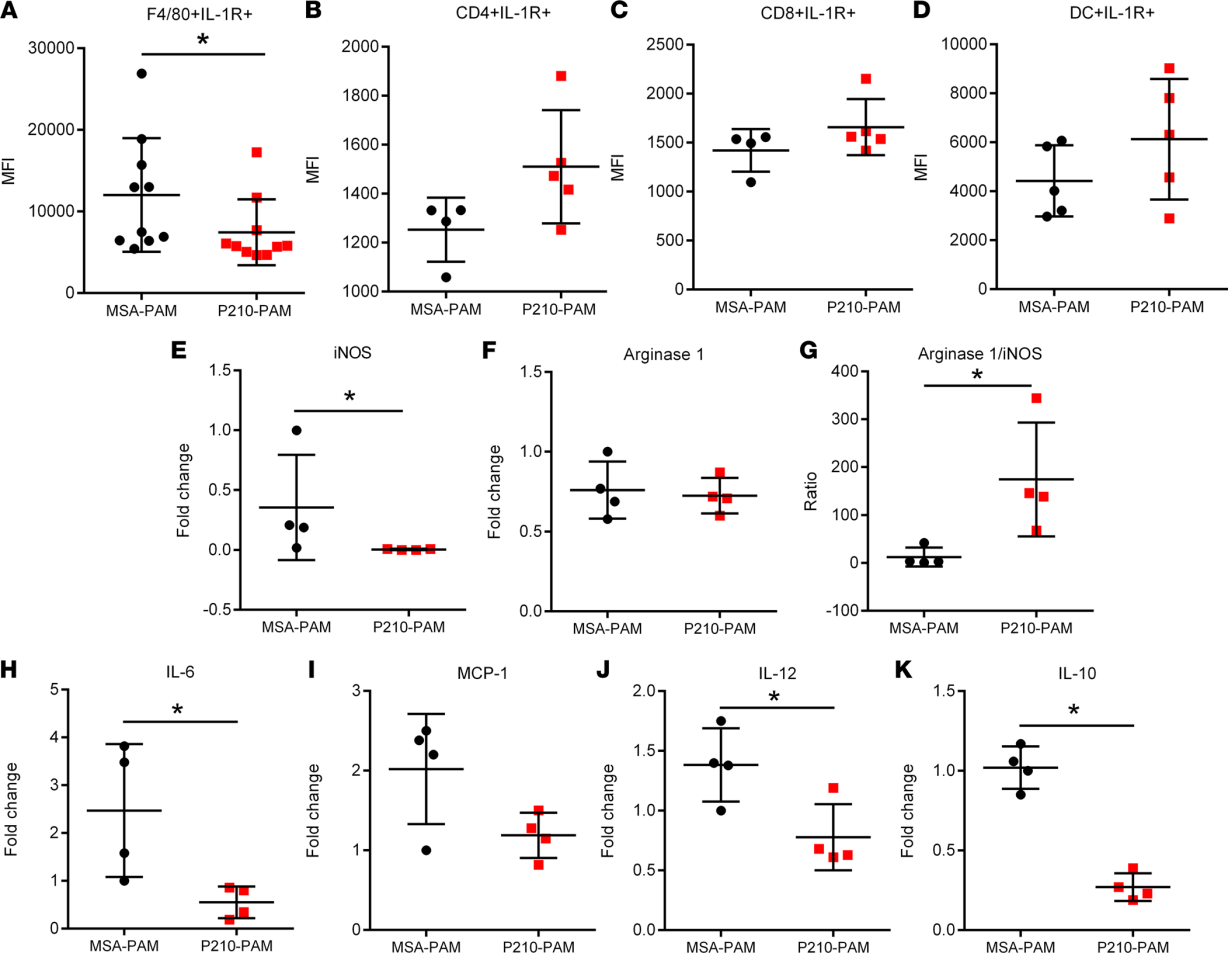
Macrophage phenotype in P210-PAM–immunized *ApoE^–/–^* mice. Splenic IL-1R1 expression measured by MFI in (**A**) F4/80^+^ monocyte/macrophage cells, (**B**) CD4^+^ T cells, (**C**) CD8^+^ T cells, and (**D**) DCs. Macrophages isolated from the peritoneal cavity of immunized mice elicited by thioglycolate injection assessed for (**E**) iNOS and (**F**) arginase 1 mRNA expression. (**G**) Ratio of arginase 1 to iNOS mRNA expression. Macrophages were further phenotyped using mRNA expression of (**H**) IL-6, (**I**) MCP-1, (**J**) IL-12, and (**K**) IL-10. Number of mice used in each group is represented by the number of dots in individual figure. *P < 0.05 Mann-Whitney except for **J** and **K**, which were analyzed by 2-tailed *t* test.

Since P210-PAM immunization elicited an antigen-specific regulation of CD4^+^ and CD8^+^ T cells, we next tested if such regulation involved the IL-1β signaling pathway given the known involvement of this pathway in atherosclerosis. There was a significant reduction in splenic IL-1 receptor type 1 (IL-1R1), IL-6, and IL-17a gene expression in P210-PAM–immunized mice but no difference in IL-1β gene expression when compared to MSA-PAM–immunized mice (Figure 5, M–P). Interestingly, the reduced IL-1R1 gene expression was primarily due to decreased expression on splenic F4/80^+^ cells but not on CD4^+^ T cells, CD8^+^ T cells, or DCs (Figure 6, A–D), suggesting modulation of macrophages by P210-PAM immunization. To delineate this pathway further, we examined the phenotypes of thioglycolate-induced peritoneal macrophages from P210-PAM– or MSA-PAM–immunized mice. The mRNA expressions of inducible NOS (iNOS), IL-6, IL-12, and IL-10 were all significantly reduced, with a trend toward decreased monocyte chemoattractant protein–1 (MCP-1), in macrophages from P210-PAM–immunized mice (Figure 6, E and H–K). Lack of difference in arginase 1 expression between the groups rendered higher arginase 1/iNOS expression ratio in macrophages from P210-PAM–immunized mice (Figure 6, F and G).

### ApoB_KTTKQSFDL_ pentamer.

The results thus far provide evidence that P210-PAM immunization provokes a response that modulates T cell function and macrophage phenotypes and reduces atherosclerosis in *ApoE^–/–^* mice, supporting the feasibility of the immunogenic nanoparticle approach to reduce atherosclerosis. However, there are limitations of antigen-based immune modulation because it depends on the propensity of specific peptides to bind and be presented as immune-antigens by class I and class II MHC. Our previous reports on P210 T cell responses in *ApoE^–/–^* mice identified MHC-I/CD8^+^ T cell signaling as a mechanism for the protective effects of P210 immunization (4, 8). An approach to bridge the experimental investigation toward translational application was therefore developed by screening class I HLA propensity to bind P210.

**Figure 7:**
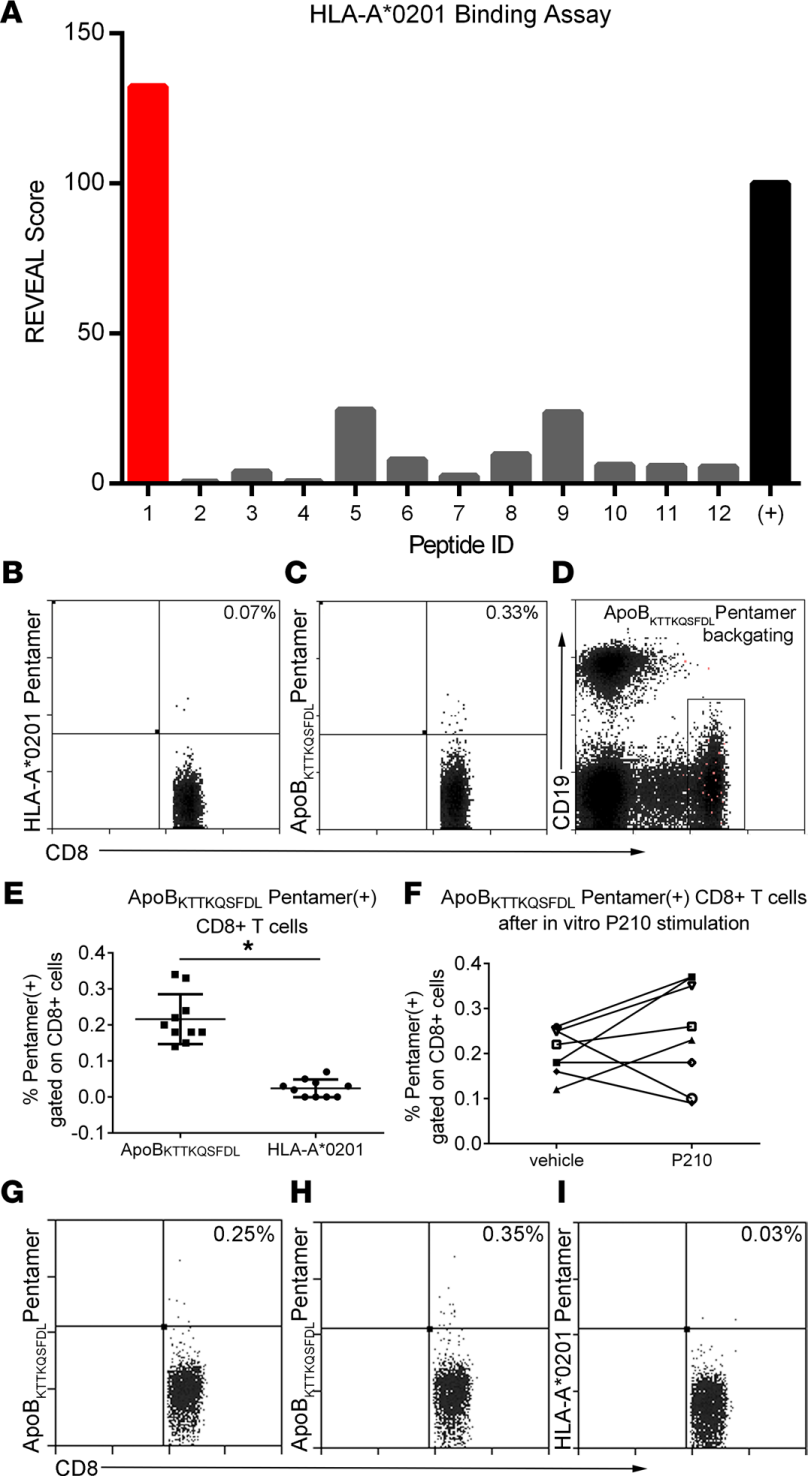
ApoB_KTTKQSFDL_ pentamer. (**A**) Binding scores of P210 epitope sequences listed in Table 2 from REVEAL binding assay. Representative plot of PBMCs from an HLA-A*02:01^+^ volunteer stained with (**B**) HLA-A*02:01 control pentamer or (**C**) ApoB_KTTKQSFDL_ pentamer, (**D**) with backgating in magenta. (**E**) ApoB_KTTKQSFDL_ pentamer^+^CD8^+^ T cells in PBMCs of HLA-A*02:01^+^ volunteers compared with control HLA-A*02:01 pentamer (*n* = 10). (**F**) Aliquots available from 8 of the same volunteers were stimulated with 20 μg/mL P210 peptide or vehicle (sterile double-distilled H_2_O) for 5 days. Representative scatterplot of vehicle (**G**) or P210 peptide (**H**) sample stained with ApoB_KTTKQSFDL_ pentamer. (**I**) The P210-stimulated samples were also stained with HLA-A*02:01 control pentamer as reference for pentamer specificity. *P < 0.05 by 2-tailed *t* test.

The human class I HLA that occurs with the highest frequency in North America is HLA-A*02:01, and P210 epitope binding to HLA-A*02:01 was tested by ProImmune using the REVEAL assay, which used 9-mer sequential peptides of P210 to assess binding to HLA-A*02:01 (Table 2). The first 9-mer scored well, comparable to the positive control (Figure 7A), suggesting that P210 contains at least 1 epitope that has the propensity to bind and potentially be presented by HLA-A*02:01; hence, a pentamer based on this 9-mer sequence (ApoB_KTTKQSFDL_ pentamer) was generated for testing. ApoB_KTTKQSFDL_ pentamer was able to detect a small but significant population of P210-specific CD8^+^ T cells in PBMCs from healthy HLA-A*02:01^+^ volunteers (Figure 7, B–E). In 5 out of 8 tested samples, culturing these PBMCs with P210 for 5 days resulted in an increase of pentamer-specific CD8^+^ T cells (Figure 7, F–I).

### A2Kb-Tg ApoE^–/–^ mice express functional chimeric A2Kb protein.

**Figure 8:**
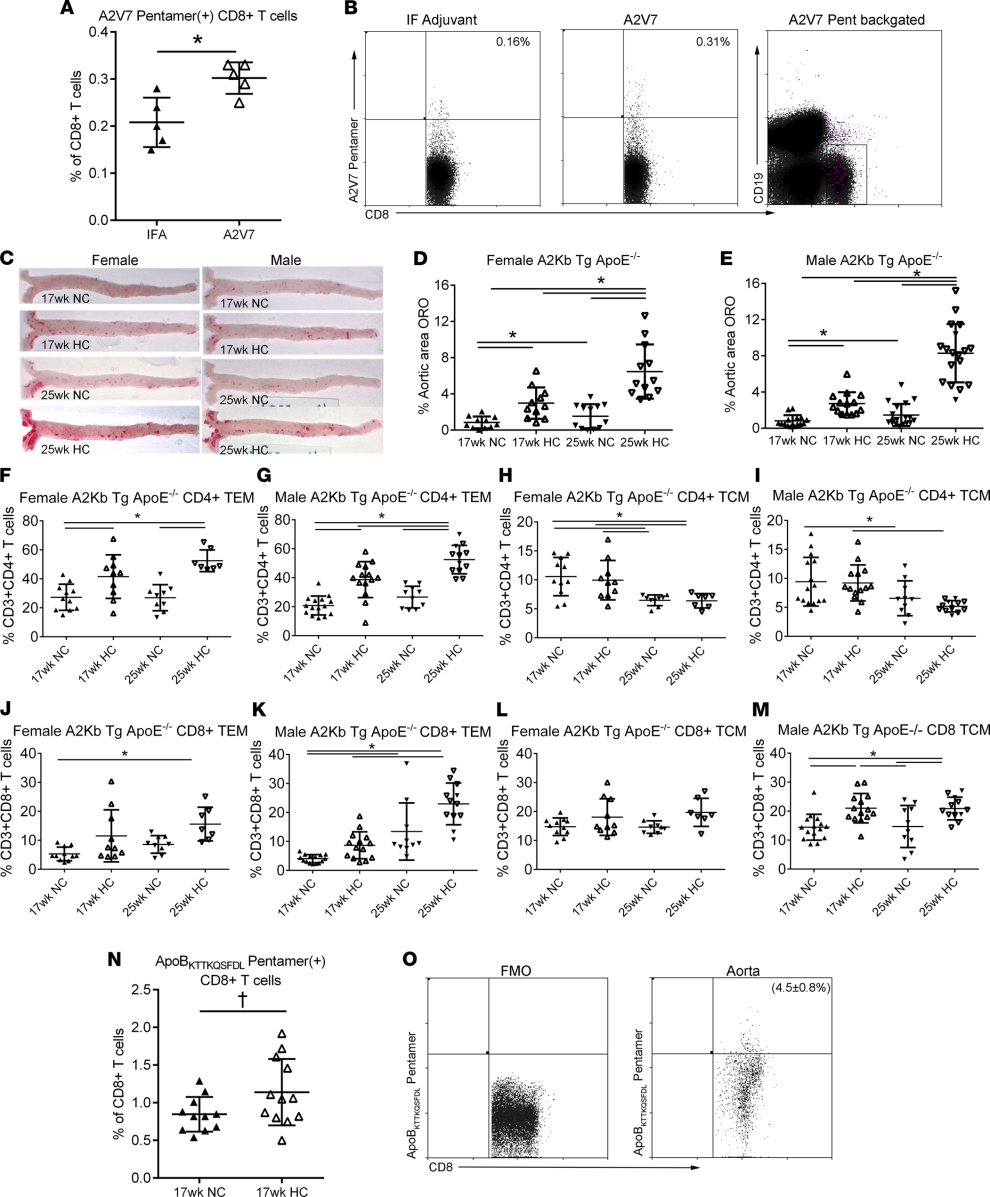
HLA-A*02:01 transgenic mouse model. (**A**) Functional test of transgene in *A2Kb-Tg ApoE^–/–^* mice immunized with A2V7 and the detection of A2V7 pentamer^+^CD8^+^ T cells. (**B**) Representative scatterplot of A2V7 pentamer^+^CD8^+^ T cells in adjuvant- or A2V7-immunized mice with backgating in magenta. (**C**) Representative photographs of oil red O–stained en face aortas from female and male *A2Kb-Tg ApoE^–/–^* mice fed normal chow (NC) or high cholesterol diet (HC) for 8 or 16 weeks starting at 9 weeks of age. (**D**) Aortic atherosclerosis in female and (**E**) male mice at 17 and 25 weeks of age. (**F**–**I**) CD4^+^ memory T cells and (**J**–**M**) CD8^+^ memory T cells in *A2Kb-Tg ApoE^–/–^* mice. (**N**) HLA-A*02:01-P210 pentamer^+^CD8^+^ T cells in splenocytes of 17-week-old *A2Kb-Tg ApoE^–/–^* mice and (**O**) in plaques of mice aged >63 weeks old after 4 weeks of HC diet feeding; *n* = 4. *T* test for 2-group comparison; 1-way ANOVA with Holm-Šidák multiple comparisons test for more than 2 groups. Number of mice in each group is represented by the number of dots in individual figure. *P < 0.05; ^†^*P* = 0.06.

The transgene construct used for developing the mouse model is described in Methods and Supplemental Methods. After obtaining *A2Kb-Tg ApoE^–/–^* offspring from breeding, immunization of male mice with an HLA-A*02:01–restricted hepatitis C virus (HCV) peptide A2V7 significantly increased A2V7-pentamer^+^ CD8^+^ T cells in the spleen (*P* < 0.05), compared with incomplete Freund’s adjuvant–injected (IFA-injected) male mice (Figure 8, A and B). The results demonstrate presentation of the HLA-A*02:01–restricted HCV peptide to activate CD8^+^ T cells, supporting the functional expression of the chimeric transgene. Colony expansion was then undertaken to characterize atherosclerosis in the chimeric model.

### High-cholesterol diet induces atherosclerosis in A2Kb-Tg ApoE^–/–^ mice.

Feeding female *A2Kb-Tg ApoE^–/–^* mice with a high-cholesterol diet for 8 weeks starting at 9 weeks of age increased aortic atherosclerosis compared with normal chow feeding (Figure 8, C and D; 17wk HC and 17wk NC, respectively). High-cholesterol diet for 16 weeks significantly increased circulating cholesterol levels (1274 ± 297 mg/dL vs. 661 ± 119 mg/dL, *P* < 0.001 by *t* test) and aortic atherosclerosis (6.5 ± 3.0% vs. 1.5 ± 1.3%; Figure 8, C and D; 25wk HC and 25wk NC, respectively) in female mice. Body weight was comparable in female mice fed the 2 different diets (Supplemental Figure 7A). Similarly, in male mice, high-cholesterol diet feeding for 8 weeks compared with normal chow significantly increased aortic atherosclerosis (Figure 8, C and E). High-cholesterol diet for 16 weeks increased circulating cholesterol levels (1760 ± 475 mg/dL vs. 617 ± 114 mg/dL, *P* < 0.001 by *t* test) and aortic atherosclerosis (8.3 ± 3.2% vs. 1.5 ± 1.2%, Figure 8, C and E). Body weight was also comparable in male mice fed the 2 different diets (Supplemental Figure 7B). Aortic sinus lesion size was also significantly increased in mice fed high-cholesterol diet compared with those fed normal chow (Supplemental Figure 7, C and D). The results show that aortic atherosclerosis burden is increased by high-cholesterol diet in both male and female transgenic mice.

### T cell profile and P210-specific T cells in A2Kb-Tg ApoE^–/–^ mice.

Feeding *A2Kb-Tg ApoE**^–/–^*** mice with high-cholesterol diet for 16 weeks significantly increased CD4^+^ effector memory T cells without change in central memory T cells in both female and male mice compared with normal chow feeding (Figure 8, F–I). CD8^+^ effector memory T cells were also significantly increased in high-cholesterol diet–fed female and male mice. However, high-cholesterol diet increased CD8^+^ central memory T cells significantly in male mice only (Figure 8, J–M).

The results thus far show that the *A2Kb-Tg ApoE^–/–^* mouse is a valid experimental model for atherosclerosis. Given that the results suggest responses are comparable between male and female mice, further analysis combined both sexes for the rest of the studies. ApoB_KTTKQSFDL_ pentamer staining showed that P210-specific CD8^+^ T cells were increased in *A2Kb-Tg ApoE**^–/–^*** mice fed a high-cholesterol diet for 8 weeks compared with mice fed normal mouse diet (Figure 8N, P = 0.06). P210-specific CD8^+^ T cells were also observed in the aortic plaque of high-cholesterol diet–fed mice by flow cytometric analysis of digested whole aortic tissue (Figure 8O). These results support the potential involvement of P210-specific CD8^+^ T cells in atherosclerosis, in agreement with our previous studies, and use of ApoB_KTTKQSFDL_ pentamer as a tool to assess P210-specific CD8^+^ T cell response in atherosclerosis.

### P210-PAM induced persistent P210-specific CD8^+^ T cells in A2Kb-Tg mice and reduced atherosclerosis.

**Figure 9:**
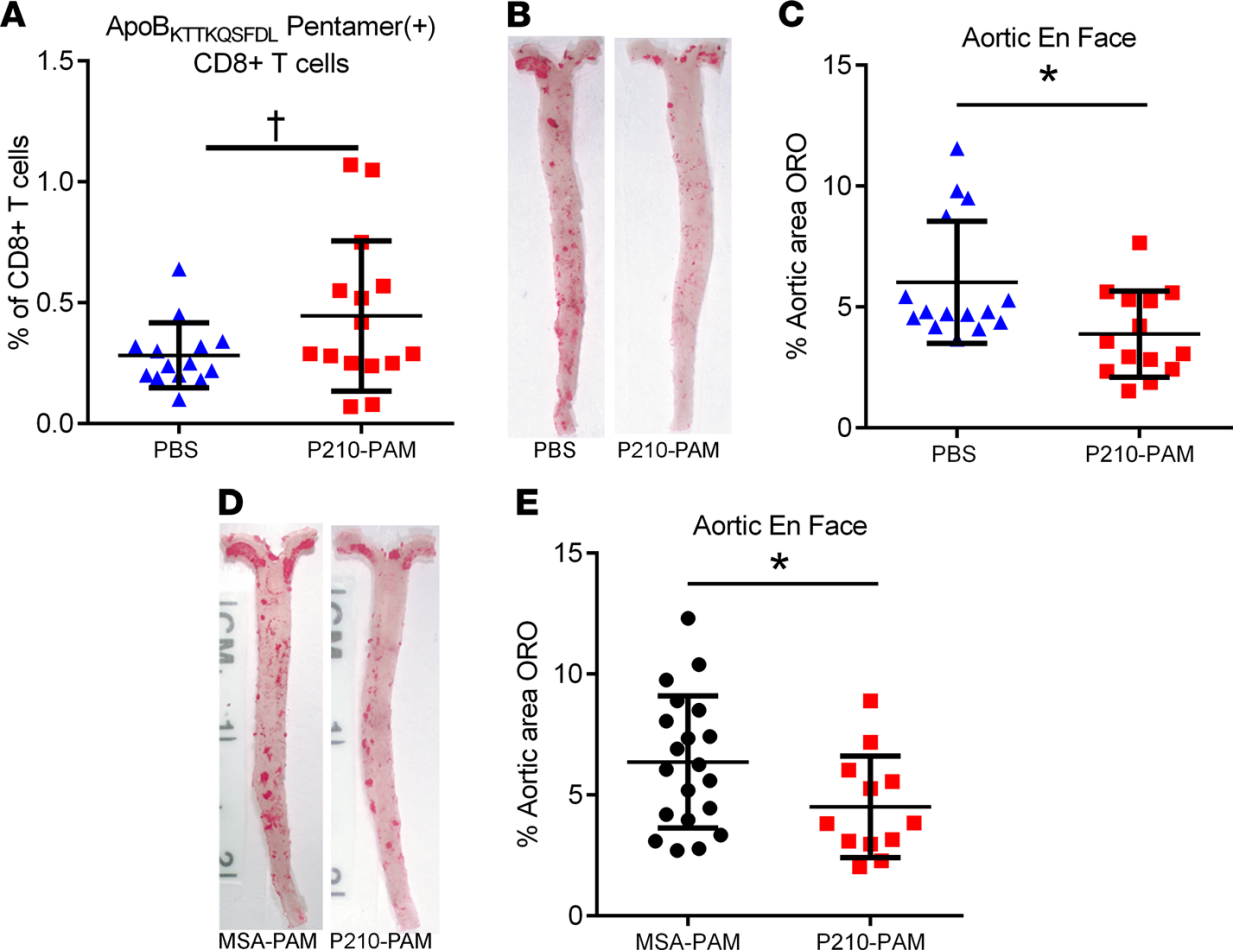
P210-PAM immunized *A2Kb-Tg ApoE^–/–^* mice. (**A**) Detection of ApoB_KTTKQSFDL_ pentamer ^+^ cells in splenocytes of *A2Kb-Tg ApoE^–/–^* mice 13 weeks after second booster injection with either PBS or P210-PAM; ^†^*P* = 0.08, 2-tailed *t* test. (**B**) Representative photographs of aortic atherosclerosis in these mice. (**C**) Measurement of percentage aortic atherosclerosis area. (**D**) Representative photographs of aortic atherosclerosis in a second cohort of mice immunized with either MSA-PAM or P210-PAM. (**E**) Percentage aortic atherosclerosis area measurement. *P < 0.05, 2-tailed *t* test.

The results thus far show the *A2Kb-Tg ApoE^–/–^* mouse is a valid humanized atherosclerosis model to investigate translational use of P210-PAM as an antigen-specific immune-modulating therapy. *A2Kb-Tg ApoE^–/–^* mice were immunized as described and were fed a high-cholesterol diet from 13 weeks of age until euthanasia at 25 weeks of age. We first tested if ApoB_KTTKQSFDL_ pentamer would detect P210-specific CD8^+^ T cells 13 weeks after the last booster injection. ApoB_KTTKQSFDL_ pentamer^+^CD8^+^ T cells were detected in splenocytes of the immunized mice, trending higher compared with control mice injected with PBS (Figure 9A, P = 0.08). Furthermore, *A2Kb-Tg ApoE^–/–^* mice immunized with P210-PAM had significantly reduced aortic atherosclerosis compared with mice injected with PBS (Figure 9, B and C). An additional group of *A2Kb-Tg ApoE^–/–^* mice were then immunized with MSA-PAM to determine if amphiphilic micelles with a different self-peptide would affect atherosclerosis in the humanized mouse model. There was no significant effect of MSA-PAM on atherosclerosis compared to PBS control, and P210-PAM–immunized mice had significantly reduced atherosclerosis compared with MSA-PAM (Figure 9, D and E). There was no difference of circulating levels of total cholesterol or LDL-C between PBS- and P210-PAM–immunized mice (Supplemental Figure 8, A and B), whereas circulating levels of total cholesterol and LDL-C in P210-PAM–immunized mice were higher than MSA-PAM–immunized mice (Supplemental Figure 8, C and D). No differences were noted in T cell and macrophage infiltration of the aortas of the immunized *A2Kb-Tg ApoE^–/–^* mice (Supplemental Figure 8, E–H). The results support P210-PAM as a viable translational immune-modulating therapy. The persistence of the P210-specific response can be assessed using a pentamer specific for an epitope of P210.

## Discussion

In this study, we report the following findings: (a) P210-specific T cell responses exist in humans with ASCVD; (b) P210 peptide can be taken up by DCs via proteoglycan binding; (c) P210, when used in a nanoparticle platform (P210-PAM), costains with MHC-I and modulates T cells in *ApoE^–/–^* mice; (d) in hypercholesterolemic *ApoE^–/–^* mice, immunization with P210-PAM dampens P210-specific CD4^+^ T cell proliferative response and CD8^+^ T cell cytolytic response, modulates macrophage phenotypes, and significantly reduces aortic atherosclerosis; (e) we successfully developed and characterized a humanized atherosclerosis mouse model with HLA-A*02:01/Kb chimera in *ApoE^–/–^* background, serving as a translational bridge to potential future human testing; and (f) most importantly, immunization with P210-PAM in the chimeric mice reduced atherosclerosis, indicating P210-PAM is a viable strategy for potential human application. Although P210 has been shown by several investigators as an effective immune modulation strategy to confer protective effect on atherosclerosis, our studies investigated its use in a nanoparticle formulation and tested it in humanized chimeric mice to demonstrate potential translational human application.

Investigations on the immune response against various ApoB-100 peptides, including P210, have demonstrated their potential use as peptide antigens for immune modulation therapies (6, 38). Although P210 humoral immune response has been demonstrated in human ASCVD (9), information on cellular immune responses against P210 in humans is lacking. One hallmark feature of antigen-experienced T cells is activation upon antigen rechallenge. Given that patients with ACS have underlying atherosclerotic vascular disease, we tested if there is a population of P210-specific T cells that can be activated upon rechallenge of P210. The AIM assay showed induction of CD69^+^CD134^+^ activation markers on CD4^+^ T cells, supporting the existence of P210-experienced T cells in humans with atherosclerosis. Similarly, we found significantly different responses of CD8^+^ effector and effector memory T cells to P210 recall stimulation in patients with ACS when compared with those from healthy volunteers (Figure 1, I and J). Thus, our data support the notion that cellular immune responses to P210 exist in human ASCVD. Although the causal role of such CD8^+^ effector memory T cell response in ASCVD remains to be elucidated, it should be noted that memory T cells are enriched in atherosclerotic plaques (39), correlated with atherosclerosis in humans and mouse models (40), and associated with plaque progression and rupture (41). These observations highlight the involvement of memory T cells in atherosclerosis. To our knowledge, this is the first study to demonstrate P210-specific cellular immune responses in human ASCVD.

It is not clear how an autoimmune response to a self-antigen like P210 is triggered. However, the lysine-rich nature of the peptide may provide some insight. A common property of CPPs is their cationic nature due to enrichment with lysine and/or arginine residues within the sequences (33, 42). CPPs interact with negatively charged cell surface heparan sulfate proteoglycans to gain cell entry (32, 33). Interestingly, part of the P210 peptide belongs to the proteoglycan-binding domain of the ApoB-100 protein (43, 44) and has been shown to be a functioning CPP to generate antigen-specific CD8^+^ T cell response (34). Our results provided experimental evidence that P210 indeed has properties of a CPP with proteoglycan-binding properties that facilitates its internalization by DCs.

We have previously demonstrated the intrinsic CD8^+^ T cell recall response to P210 stimulation in naive hypercholesterolemic mice (8). However, it is unknown if the immunologic property of P210 changes when formulated as PAM nanoparticles. We first demonstrated that DCs can uptake P210-PAM and that P210 (or its fragment) costains with MHC-I using confocal microscopy. Our observation that P210-PAM immunization increased CD4^+^CD25^+^FoxP3^+^ and CD8^+^CTLA-4^+^ T cells suggested an induction of regulatory CD4^+^ and CD8^+^ T cells. This was further confirmed by functional experiments showing antigen-specific reduction of CD4^+^ T cell proliferative response and CD8^+^ cytotoxic T cell response to P210. More importantly, P210-PAM immunization significantly reduced aortic atherosclerosis in mice when compared with control groups (Figure 9, B and C).

A notable observation is that P210-PAM immunization, in addition to modulating T cells, modulates macrophages. Interaction between T cells and monocytes/macrophages has been previously reported. CD8^+^ T cells promote BM monocyte production via an IFN-γ–mediated mechanism in viral infection (45). Depletion of CD8^+^ T cells reduced atherosclerosis, decreased the number of mature monocytes in the BM and spleen of hypercholesterolemic mice, and reduced GM-CSF and IL-6 expression in BM cells but did not affect the recruitment of monocytes to atherosclerotic plaques (46). In tissues from obese mice, activated CD8^+^ T cells differentiated peripheral blood monocytes into macrophages (47). CD4^+^CD25^+^FoxP3^+^ T cells have been shown to induce alternatively activated monocytes with reduced inflammatory phenotype (48). Taken together, our data support the notion that P210-PAM elicits an interaction between T cells and macrophages and reduces the immune-inflammatory responses in atherosclerosis at the level of both innate and adaptive immunity.

The physicochemical properties of nanoparticles play a vital role in determining the immune responses of nanoparticle-based vaccines. Nanoparticles 20–200 nm in diameter are usually internalized by APCs to elicit T cell response. Cationic nanoparticles with positive charges facilitate lysosomal escape and cross-presentation to MHC-I (49). Solid-core nanoparticles with antigen on the surface elicit stronger CD8^+^ T cell response whereas polymersomes with antigen incorporated inside the core bias toward CD4^+^ T cell responses. This differential immune response based on physicochemical properties is not strictly dichotomous as reported data have shown solid-core nanovaccines can also induce CD4^+^ T cell response (50). Although cylinder-shaped P210-PAM with P210 on its surface would be predicted to skew toward a CD8^+^ T cell response, our data indicate that P210-PAM elicits regulatory responses in both CD4^+^ and CD8^+^ T cells. The concept of delivering autoantigens by nanoparticles to modulate autoimmune diseases has been tested before. The severity of autoantigen-induced experimental autoimmune encephalomyelitis or type 1 diabetes can be reduced by this approach (51, 52). The beneficial effect was thought to be mediated by promoting differentiation of disease-primed autoreactive CD4^+^ T cells into T_R_1-like cells or by expanding memory-like antidiabetogenic CD8^+^ T cells, respectively (51, 52). Given that P210 is potentially an atherogenic autoantigen, the induction of regulatory T cell responses by P210-PAM is consistent with this view. It should be noted that peptide-loaded MHC-II or MHC-I complex was a part of nanoparticles used by Clemente-Casares et al. (51) and Tsai et al. (52), whereas the P210-PAM in this study did not contain MHC molecules.

The mean reduction of atherosclerosis by P210-PAM immunization in the current study was 42% and 37% in *ApoE^–/–^* mice and *A2Kb-Tg ApoE^–/–^* mice, respectively, consistent with the mean reduction of atherosclerosis between 25% and 60% reported by investigators using different formulations (4–7, 11, 53–55). Although the reported atheroreduction effect from using various P210 formulation has been consistent across different studies, the reported immune responses to P210 differ. Some reported atheroreduction was associated with increased P210-related antibody production (5–7); some reported induction of regulatory T cell responses (5, 11, 56). Nevertheless, the reported data support the notion that P210 is capable of eliciting multiple humoral and cellular immune responses, though each study used different doses, preparations, and delivery methods of P210.

A few studies have addressed the immune mediators for the atheroreduction effect produced by P210 immunization. Rattik et al. showed B cells pulsed with CTB-P210 reduced atherosclerosis after being transferred into naive recipients (7), but it is not clear if the B cells functioned as APCs or antibody-producing cells induced by peptide-pulsing. Another study showed that a P210 IgG antibody preparation from rabbits was able to reduce murine atherosclerosis in a passive immunization fashion (57). We previously reported P210 immunization was able to mount antibody response and a CD8-biased T cell response (4). Using a cell transfer strategy, we demonstrated that CD8^+^ T cells, not B cells or CD4^+^CD25^+^ T cells, were the mediators responsible for the atheroprotective effect of P210 immunization (4).

The involvement of P210-specific CD8^+^ T cells described above prompted our investigation to transition toward translational studies. The first step to potentially translate our immunization strategy for clinical testing is to establish if this immunization strategy can elicit immune response in humans. To achieve this, it is necessary to develop tools and models to detect antigen-specific T cells and for preclinical endpoint testing, respectively. An HLA-A*02:01-based P210-related pentamer, named ApoB_KTTKQSFDL_ pentamer, was generated to track P210-specific CD8^+^ T cells as a marker for cellular immune response. Using this pentamer, we demonstrated the existence of a small but significant number of antigen-specific CD8^+^ T cells that responded to P210 rechallenge in human PBMCs. We also generated an animal model with a prevalent human MHC-I allele, HLA-A*02:01, to produce proof-of-concept data before advancing this strategy to human testing. We chose HLA-A*02:01 as a representative human MHC-I allele due to its high frequency in the population and generated a new animal model with transgenic expression of human HLA-A*02:01 in *ApoE^–/–^* mice on a *C57BL/6J* background. These mice mounted antigen-specific CD8^+^ T cell response to the CD8-restricted peptide A2V7 from human HCV as assessed by pentamer after immunization, indicating functional HLA-A*02:01 allele. With P210-PAM immunization, these mice elicited higher splenic ApoB_KTTKQSFDL_ pentamer^+^CD8^+^ T cells when compared with nonimmunized mice. P210-PAM immunization significantly reduced aortic atherosclerosis when compared with control groups, supporting the potential use of P210-PAM for human testing. Given the same genetic background between *ApoE^–/–^* and chimeric mice, we speculate P210-PAM immunization modulates macrophages and CD4^+^ and CD8^+^ T cells in *A2Kb-Tg ApoE**^–/–^*** mice similarly to *ApoE^–/–^* mice. However, this remains to be confirmed.

The concept of using active immunization strategies to reduce atherosclerosis has progressed in the past 3 decades. The search for suitable antigens has evolved from using the whole LDL molecule as an antigen to subunits of lipoprotein, such as ApoB-100 peptides. In murine atherosclerosis, immune responses to LDL or its related ApoB-100 peptides are present, and modulation of such responses by active immunization with LDL or ApoB-100 peptides confers atheroprotective effects. If the same analogy applies to humans, given the existence of immune responses to LDL or ApoB-100 peptides in humans, we hypothesize similar atheroprotective effect from active immunization in humans. Here, we demonstrate physicochemical and immunological properties of P210-PAM and its effects on T cell responses and atherosclerosis, supporting the use of P210-PAM as an immune modulation strategy targeting atherosclerosis. Such nanoparticle platforms are suitable for human application as nanoparticle-based vaccines are now in clinical use. More importantly, our successful use of P210-PAM in chimeric mice with human MHC-I allele provided proof-of-concept data showing potential efficacy in human immune system and paved the way for future testing in humans.

## Methods

### Human PBMCs.

The protocols were approved by the Cedars-Sinai Institutional Review Board (IRB). PBMCs were isolated from blood collected from 13 patients with ACS within 72 hours of admission to the Cardiac Intensive Care Unit at Cedars-Sinai Medical Center. Patients provided consent under the approved IRB protocol Pro48880. Exclusion criteria were inability to give informed consent, age less than 18 years old, active cancer treated with chemotherapy or radiation, taking immune-suppressive drugs, and pregnancy. The data collected were limited to age, sex, LDL levels, and use/nonuse of cholesterol-lowering medication (Table 1). PBMCs were isolated using Ficoll (GE Healthcare) density gradient centrifugation and cryopreserved in commercially available cryogenic solution (Immunospot) in liquid nitrogen. Cryopreserved PBMCs from healthy controls (*n* = 14) were purchased from a commercial source (Immunospot).

### AIM assay in human PBMCs.

Cryopreserved PBMCs were thawed, rinsed in antiaggregation solution (Immunospot), and seeded in culture plates at a density of 3 × 10^6^ cells/mL in RPMI 1640 medium (Thermo Fisher Scientific) supplemented with 10% heat-inactivated pooled human serum (Innovative Research, Inc) and 1× antibiotic/antimycotic (Gibco). After resting for 4 hours, cells were preincubated with 0.5 mg/mL anti-CD40 antibody for 15 minutes, then stimulated with 20 μg/mL P210 peptide, 0.5× T cell stimulation cocktail containing PMA and ionomycin (Thermo Fisher Scientific), or CMV (pp65) Peptide Pool (STEMCELL Technologies) as a nonrelevant antigen control, whereas cells without treatment served as nonstimulated control. Cells were harvested 16 hours after seeding, stained for viability (LIVE/DEAD Fixable Aqua Dead Stain Kit, Thermo Fisher Scientific), and subjected to cell surface staining for flow cytometry (BD LSRFortessa Cell Analyzer) using the following antibodies: CD4, CD8, CD25, CD69, OX40 (CD134), CD137 (4-1 BB), and CD154 (CD40L). Isotypes were used as staining control, and eFluor506-labeled CD14, CD16, and CD19 antibodies were used as dump staining to exclude B cells, DCs, macrophages, granulocytes, eosinophil cells, and neutrophil cells. The results are expressed as fold change (ratio between the signal in the antigen-stimulated condition and the signal in the unstimulated condition) for each participant, consistent with the reported AIM assay (28). Antibodies used in the AIM assay are listed in Supplemental Table 1.

### Peptide stimulation of human PBMCs.

Cryopreserved PBMCs were thawed, rinsed, and cultured as in the AIM assay but without resting. Cells were stimulated with 20 μg/mL P210 peptide or 0.5× T cell stimulation cocktail containing PMA and ionomycin (Thermo Fisher Scientific) with nontreated cells serving as negative control. Culture medium was added at one-third of the starting volume 48 hours later to replenish the nutrients in the medium. Cells were harvested 72 hours after seeding, stained for viability (LIVE/DEAD Fixable Aqua Dead Stain Kit), and subjected to cell surface staining for flow cytometry using the following antibodies: CD3, CD4, CD8, CD45RA, CD45RO, CD62L, and CD197 (CCR7). Isotypes were used as staining control. CD4^+^ or CD8^+^ T effector cells were gated on CD45RO^+^CD62L^–^CD197^–^. T effector memory cells were CD45RO^+^CD45RA^–^CD62L^–^CD197^–^. Antibodies used are listed in Supplemental Table 2. Results were tabulated as response index using the following calculation: (% peptide stimulation – % no stimulation)/(% cocktail stimulation) × 100.

The results are expressed as response index to account for inherent variations introduced by culturing cells in vitro over time, controlled for by assessing response relative to baseline cell phenotype (% no stimulation) and maximal stimulation (% cocktail stimulation) for each patient’s PBMCs (58). Each data point represents 1 patient.

### Animals.

All mice were maintained under standard animal housing conditions with a 12-hour light/12-hour dark cycle and were fed ad libitum with a regular chow diet (5015, PMI Nutrition International) unless mentioned otherwise. All animal procedures were done in compliance with NIH guidelines and were approved by the IACUC. *B6.129P2-Apoe^tm1Unc^/J* (*ApoE^–/–^*) mice were purchased from The Jackson Laboratory (stock 002052). A2Kb-transgenic CB6F1-Tg(HLA-A*02:01/H2-Kb)A*0201 mice were purchased from Taconic Biosciences (model 9659).

### Amphiphile synthesis, assembly, and characterization.

See Supplemental Methods for details.

### DC uptake of FITC-labeled P210 and P210-PAM.

P210 peptide was labeled with FITC using a commercially available kit (Thermo Fisher Scientific). To prepare FITC-P210-PAM, P210 peptide was first labeled with FITC on the last lysine on the C-terminal when the peptide was synthesized; then the labeled P210-FITC was assembled into FITC-P210-PAM using methods described in the Supplemental Methods for micelle assembly.

BMDCs were prepared using BM cells from femurs and tibiae of male *ApoE^–/–^* mice. After depletion of erythrocytes with lysis buffer, BM cells were cultured in 10 cm dishes with 10 mL complete RPMI 1640 medium supplemented with 20 ng/mL GM-CSF (R&D Systems) and 10 ng/mL IL-4 (Invitrogen). On day 2, 10 mL fresh culture medium was added to each dish; then 10 mL medium was replaced with fresh medium on days 4 and 6. On day 8, nonadherent, immature DCs were harvested into new culture medium containing 100 μg/mL P210-FITC or FITC-P210-PAM in complete RPMI 1640 medium. After a 4-hour incubation for P210-FITC or 6-hour incubation for FITC-P210-PAM, cells were collected and stained with antibodies against CD11c (clone N418, Invitrogen) or CD11c and H2-K^b^ (clone AF6-88.5.5.3, Invitrogen), respectively. Cells were washed and fixed in 4% paraformaldehyde followed by washing and staining with the fluorescent nuclear stain Hoechst 33342 (Thermo Fisher Scientific) or DAPI (Invitrogen). Washed cells were then smeared on a slide, briefly air-dried in the dark, and fixed in cold acetone. Photographs were then taken on a Leica or Zeiss confocal microscope visualized with liquid fluorescent mounting medium. Untreated DCs were collected and smeared on slides, air-dried, and then stained with Giemsa staining reagent (Beckman Coulter) according to the kit instructions, with photos taken using a light microscope to demonstrate dendrites.

For flow cytometric experiments, P210-FITC uptake was assessed after a 2-hour incubation and staining for CD11c. For heparan binding experiments, 100 μg/mL P210-FITC was preincubated with 100 U/mL heparan for 30 minutes at room temperature and centrifuged at 1000*g* for 5 minutes. The supernatant was carefully removed and added to the cell culture. Cells were collected after 2 hours and stained for CD11c for flow cytometry. In a separate experiment, DCs were treated with pNP-xyl, a competitive inhibitor of heparan sulfate chain addition, for 18 hours at a final concentration of 3 mM. DCs were then incubated with P210-FITC for 2 hours, collected, and stained with anti-CD11c (N418) for flow cytometry.

### PAM biodistribution in vivo.

The in vivo biodistribution of P210-PAM or MSA-PAM was evaluated by injecting 1 mM cy7-labeled PAMs in 100 μL volume subcutaneously into the scruff of the neck in *C57BL/6J* mice (*n* = 4, The Jackson Laboratory). After injection, mice were shaved and imaged over 7 days (168 hours) using an AMI HTX imaging system (Spectral Instruments). A separate group of mice was euthanized 48 hours after injection to harvest injection sites for immunostaining.

### T cell immune response to P210-PAM in naive hypercholesterolemic mice.

Splenocytes were collected from 25-week-old *ApoE^–/–^* mice euthanized after 16 weeks of high-cholesterol diet feeding consisting of 0.15% cholesterol and 21% fat (TD.88137, Envigo). RBC-lysed splenocytes were incubated with 20 μg/mL P210-PAM in complete RPMI 1640 medium for 48 hours, then stained with CD3e (clone 145-2C11, eBioscience), CD4 (clone GK1.5, BD Bioscience), CD8b (clone H35-17.2, eBioscience), CD44 (clone IM7, eBioscience), and CD62L (clone MEL-I4, eBioscience) antibodies for T effector/memory cell profiling using flow cytometry.

### Immunization with P210-PAM and phenotyping atherosclerotic lesions.

*ApoE^–/–^* mice, 7 weeks old, fed normal chow, received a subcutaneous injection of one of the following: P210-PAM, MSA-PAM, or PBS. The PAM dose used was 100 μg/mouse. Booster injections were administered at 10 and 12 weeks of age. Some mice were euthanized 1 week after the second booster for immune profiling. The rest of the mice were fed a high-cholesterol diet for 12 weeks and euthanized at 25 weeks of age. Whole aortas were cleaned, processed, and stained with oil red O to assess the extent of atherosclerosis en face. Frozen heart bases embedded in OCT (Tissue-Tek) were cryosectioned starting from the appearance of 3 complete aortic valves. Three slides with 2 sections on each slide at 4- to 5-slide intervals were grouped for aortic sinus histomorphometry. Plaque sizes and lipid content were assessed by oil red O staining using standard protocol. Macrophage presence in atherosclerotic lesions in the aortic sinus was assessed by immunohistochemistry staining with anti-CD68 (clone FA-11, BioLegend) antibody, followed with incubation with an appropriate secondary antibody (Invitrogen catalog A18869) using standard protocol. Computer-assisted morphometric analysis was performed under a blinded protocol by an observer using ImagePro Plus (version 4.0, Media Cybernetics Inc.). Serum levels of total cholesterol, LDL-C, and HDL-C were measured using commercially available kits according to manufacturer’s instruction (Wako).

### ELISA for P210 antibodies.

Flat-bottomed, 96-well, polystyrene plates (MaxiSorp) were precoated with 100 μL P210 (20 μg/mL) in Na_2_CO_3_-NaHCO_3_ buffer (pH 9.6) overnight at 4°C to assess antibody levels using standard protocol. The coating concentration and serum dilution were optimized in pilot experiments. Goat anti-mouse HRP-IgG (Pierce), IgM (Southern Biotech), rat anti-mouse IgG1-HRP (Invitrogen), and goat anti-mouse IgG2b-HRP (Southern Biotech) were used as detecting antibodies; and the bound antibodies were detected by developing in ABTS (Southern Biotech) as substrate; and OD values were recorded at 405 nm. Given there is no purified P210 antibody that can be used for standardization, OD of individual mice in each group was normalized against the mean OD from the PBS group and presented as adjusted OD in the figures.

### Immune profile of P210-PAM–immunized mice.

Splenocytes of immunized *ApoE^–/–^* mice that were euthanized at 13 weeks of age (1 week after second booster) were subjected to RBC lysis. An aliquot of splenocytes was stained for CD4 (GK1.5, BD Bioscience), CD8 (YTS156.7.7, BioLegend), CD25 (PC61.5, eBioscience), CTLA-4 (UC10-4B9, BioLegend), FoxP3 (R16-715, BD Bioscience), and PD-1 (29F 1A12, BioLegend) and analyzed by flow cytometry excluding nonviable cells. A second aliquot was used to assess cytolytic activity using CD107a (1D4B) staining. Briefly, splenocytes were incubated in complete RPMI 1640 medium with 2.5 μg/mL fluorescent CD107a antibody and 5 μg/mL P210 for 1 hour. Monensin (1×) was added and the cells were incubated for another 4 hours. Cells were then collected and stained with fluorescent CD3e (145-2C11, BD Pharmingen) and CD8b (H35-17.2, Invitrogen) antibodies. The cells were analyzed by flow cytometry excluding nonviable cells. T cell proliferation was assessed using BrdU. Briefly, splenocytes were cultured in complete RPMI 1640 medium at 2.5 × 10^6^ cells/mL and stimulated with P210 (20 μg/mL). Cells stimulated with Concanavalin A (2.5 μg/mL, MilliporeSigma) served as positive controls. Untreated cells served as baseline controls. After 48 hours, BrdU was added at a final concentration of 10 μM. Cells were collected after 24 hours and stained for CD3e (BM10-37, BD Biosciences), CD4 (GK1.5, BD Biosciences), CD8b (H35-17.2, Invitrogen), and BrdU (3D4, BD Pharmingen) according to the manufacturer’s instructions (BrdU Flow Kit, BD Pharmingen), then analyzed by flow cytometry. Proliferation index was calculated as [(%BRDU^+^ cells in P210 peptide stimulation – %BRDU^+^ cells in no stimulation)/(%BRDU^+^ cells in Con A stimulation)] × 100.

### Induction of peritoneal macrophages.

*ApoE^–/–^* mice, 7 weeks old, fed normal chow, were immunized as previously described. At 13 weeks of age (1 week after second booster), mice received a peritoneal injection of 1 mL 3% thioglycolate medium (MilliporeSigma) (in PBS), and cells from the peritoneal cavity were harvested 72 hours after injection. Cells were seeded into culture dishes and incubated at 37°C for 4 hours to obtain attached peritoneal macrophages.

### Quantitative PCR.

Total RNA was extracted from spleens or peritoneal macrophages enriched from peritoneal exudate by preattaching to culture plates using TRIzol (Thermo Fisher Scientific). cDNA synthesis and quantitative real-time PCR were then performed using SuperScript VILO cDNA Synthesis Kit (Thermo Fisher Scientific), and iTaq Universal SYBR Green Supermix and iQ5 Real-Time PCR Detection System (Bio-Rad), respectively, per manufacturers’ protocols. GAPDH served as the reference gene, and results were expressed as fold change relative to nontreated cells of each sample using the ΔΔCt method. Primer sequences used for quantitative PCR are listed in Supplemental Table 3.

### Detection of ApoB_KTTKQSFDL_ pentamer^+^ CD8^+^ T cells in human PBMCs.

ProImmune was contracted to screen for potential binding epitopes in P210 to HLA-A*02:01. First, 9-mer sequence in P210 was found to have high binding score, and an HLA-A*02:01 pentamer based on this 9-mer sequence, named ApoB_KTTKQSFDL_ pentamer, was then purchased from ProImmune. For pentamer staining, commercially available HLA-A*02:01 typed cryopreserved PBMCs (Immunospot) were thawed, rinsed in antiaggregation solution (Immunospot), and divided into 2 × 10^6^ cell aliquots. ApoB_KTTKQSFDL_ pentamer staining was performed according to manufacturer’s instruction, with the HLA-A*02:01 Negative Pentamer (ProImmune) as negative control. Each sample stained for ApoB_KTTKQSFDL_ pentamer had its corresponding negative control stain. Cells were washed and then stained for CD8 (LT8) and CD19 (HIB19). Cells were again washed after staining and resuspended in 1% paraformaldehyde in 1% BSA/0.1% sodium azide and analyzed. ApoB_KTTKQSFDL_ pentamer^+^ cells for each sample were determined based on the corresponding negative pentamer.

### A2Kb-Tg ApoE^–/–^ mice.

*A2Kb-Tg ApoE^–/–^* mice were generated as briefly described below (see Supplemental Methods for details). A 3867 bp, full-length, chimeric *A2Kb* gene was cloned into pCR-XL-TOPO T vector (Thermo Fisher Scientific), and the amplified recombinant plasmids containing *A2Kb* gene were digested with restriction enzymes to yield approximately 3.9 kb fragments containing the chimeric *A2Kb* gene for fertilized *ApoE^–/–^* egg microinjection by the Cedars-Sinai Rodent Genetics Core. Germline-transmitted A2Kb chimeras obtained were screened by PCRs detecting HLA-A*02:01 fragments and flow cytometric analysis of *A2Kb* protein expression on the surface of PBMCs.

A transgenic *ApoE^–/–^* male mouse was identified and crossbred with female *ApoE^–/–^* mice. The *A2Kb*-transgenic offspring selected by flow cytometric analysis of chimeric A2Kb protein expression on peripheral blood cells were used for further breeding or experiments.

### Functional expression of A2Kb transgene.

Male *A2Kb-Tg ApoE^–/–^* mice were immunized with the HLA-A*02:01–restricted peptide A2V7 from human HCV (HCV NS5a 1987-1995, VLSDFKTWL; ProImmune) emulsified in IFA (MP Biomedicals) at 9 and 10 weeks of age by subcutaneous injection at a dose of 20 μg/100 μL. Mice immunized with 100 μL IFA alone served as control. Mice were euthanized at 11 weeks of age. HLA-A*02:01–restricted antigen-specific immune response was evaluated by flow cytometric analysis of splenocytes stained with CD19 (6D5, BioLegend), CD8α (KT15, GeneTex), and PE-conjugated HLA-A*02:01/A2V7 pentamer (ProImmune).

### Atherosclerosis in A2Kb-Tg ApoE^–/–^ mice.

*A2Kb-Tg ApoE^–/–^* mice were divided into 2 groups and fed normal chow or high-cholesterol diet starting at 9 weeks of age until euthanasia at 17 or 25 weeks of age. RBC-lysed splenocytes were stained for T effector/memory cell profile.

Another cohort of high-cholesterol diet–fed mice were euthanized at 17 weeks of age and the splenocytes stained with CD19 (6D5), CD8α (KT15), and PE-conjugated ApoB_KTTKQSFDL_ pentamer. A third cohort of female *A2Kb-Tg ApoE^–/–^* mice aged 66–68 weeks were fed a high-cholesterol diet for 4 weeks and euthanized to collect whole aortas for enzymatic digestion with 0.25 mg/mL collagenase, 0.125 mg/mL elastase, and 60 U/mL hyaluronidase (MilliporeSigma) in sterile RPMI 1640 medium for 20 minutes at 37°C. Single-cell suspensions were then stained for ApoB_KTTKQSFDL_ pentamer and flow cytometric analysis as described above.

### Immunization with P210-PAM in A2Kb-transgenic mice.

The first cohort of *A2Kb-Tg ApoE^–/–^* mice received either PBS or P210-PAM according to the same immunization protocol described earlier for *ApoE^–/–^* mice. Mice were sacrificed at 25 weeks of age, and splenocytes were subjected to flow cytometric analysis of ApoB_KTTKQSFDL_ pentamer^+^CD8^+^ T cells and their aortas subjected to morphometric analysis of oil red O^+^ plaques. To have a proper control for P210-PAM immunization, a second cohort of *A2Kb-Tg ApoE^–/–^* mice was immunized with MSA-PAM or P210-PAM using the same protocol, and aortas were analyzed for oil red O^+^ plaques.

### Statistics.

Data are presented as mean ± SD. Number of animals in each group and statistical methods are listed in text, figures, or figure legends. *P* ≤ 0.05 was considered as statistically significant, but trending data were also noted.

### Study approval.

The protocols for human studies were approved by the Cedars-Sinai IRB with patients providing written informed consent under the approved IRB protocol Pro48880. Exclusion criteria were inability to give informed consent, age less than 18 years old, active cancer treated with chemotherapy or radiation, taking immune-suppressive drugs, and pregnancy. Animal protocols were approved by the IACUC at Cedars-Sinai Medical Center, and all animal procedures were done in compliance with NIH guidelines.

## Author contributions

KYC, XZ, JZ, PCD, NTT, and EJC contributed to designing experiments, analyzing and interpreting data, and writing and revising the manuscript. XZ, JZ, NWML, and NTT contributed to conducting experiments and acquiring data. NTT and EJC provided nanoparticle vaccines for the studies. BC contributed to establishing the IRB protocol, acquiring human PBMCs, and writing and revising the manuscript. PKS contributed to the conceptualization of the study, interpretation of the data, and writing and revising of the manuscript.

## Supplementary Material

Supplemental data

## Figures and Tables

**Table 1:**
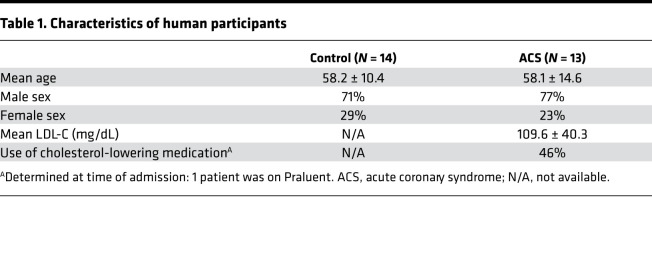
Characteristics of human participants

**Table 2:**
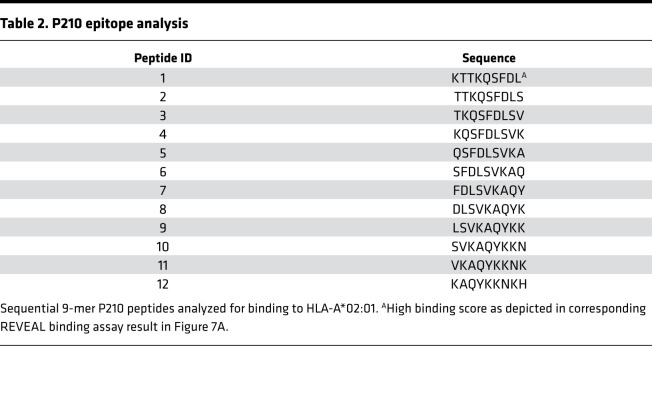
P210 epitope analysis

## Version 1 05/10/2022

In-Press Preview

## Version 2 06/08/2022

Electronic publication
