# Supplementary material for: Immunization using ApoB-100 peptide–linked nanoparticles reduces atherosclerosis
Source: JCI Insight. 2022 Jun 8;7(11):e149741. doi: 10.1172/jci.insight.149741 (PMC9220835; doi:10.1172/jci.insight.149741)
Supplement: Supplemental data [file jciinsight-7-149741-s012.pdf]

Supplementary Figures

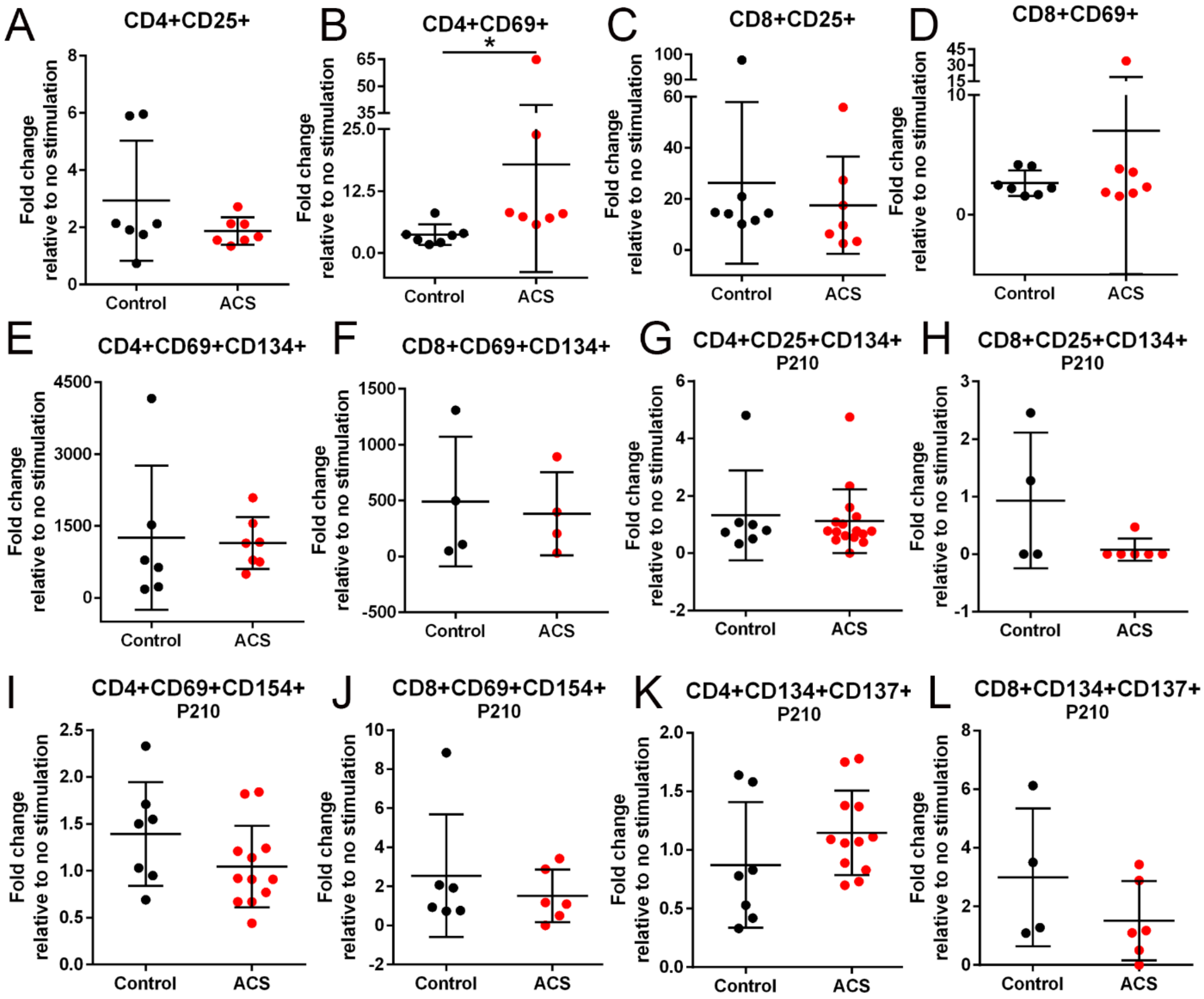

Supplementary Figure 1: (A-F) Stimulation of human PBMCs with 0.5x PMA/ionomycin cocktail served as positive control for the AIM assay. *$P<0.05$ Mann-Whitney. (G-L) Markers for other AIM(+) cells were not different compared to Controls after P210 stimulation.

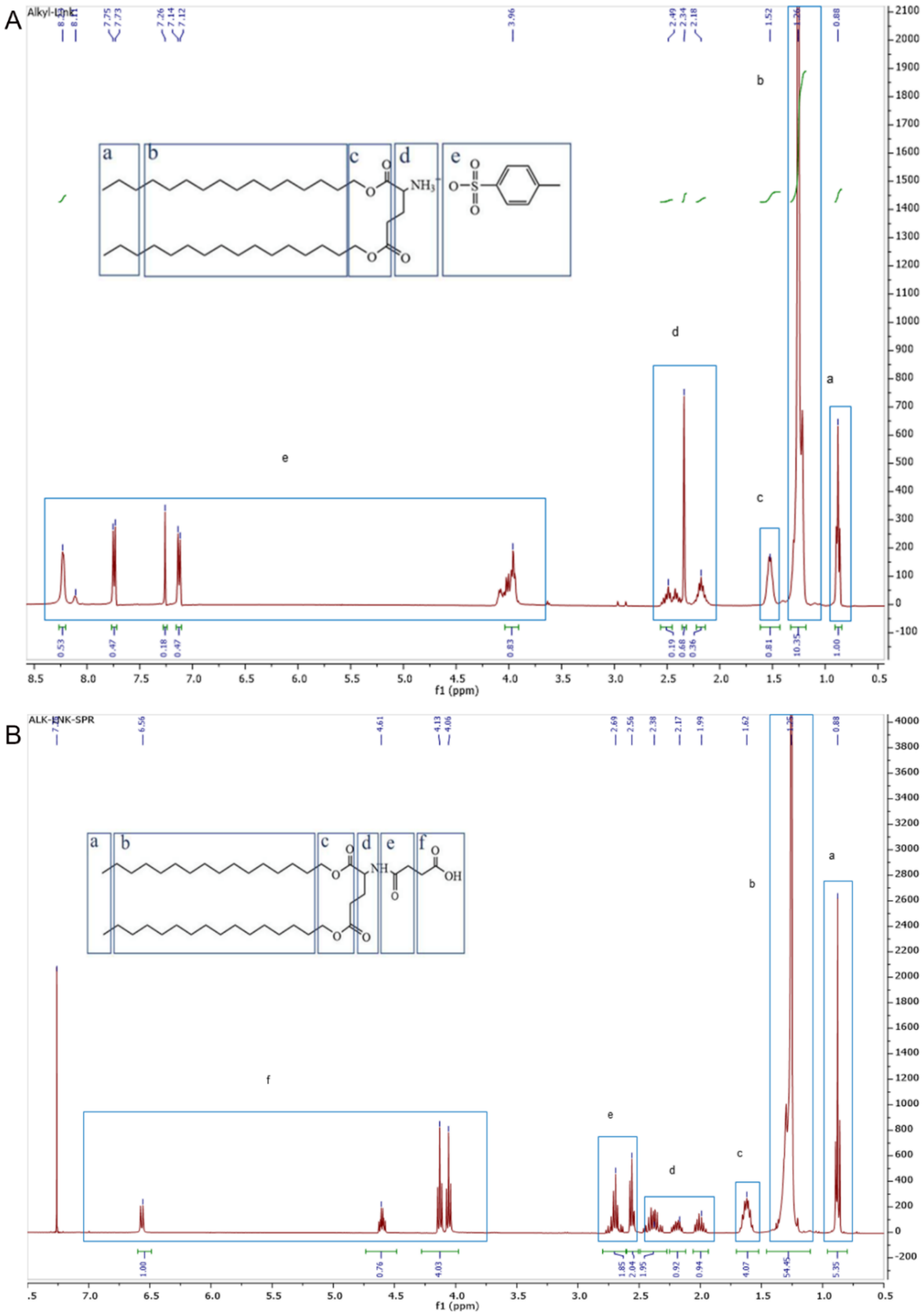

Supplementary Figure 2: ^{1}H-NMR analysis of 1’-3’-dihexadecyl L-glutamate (A) and diC$_{16}$ (1’-3’-dihexadecyl N-succinyl-L-glutamate, B).

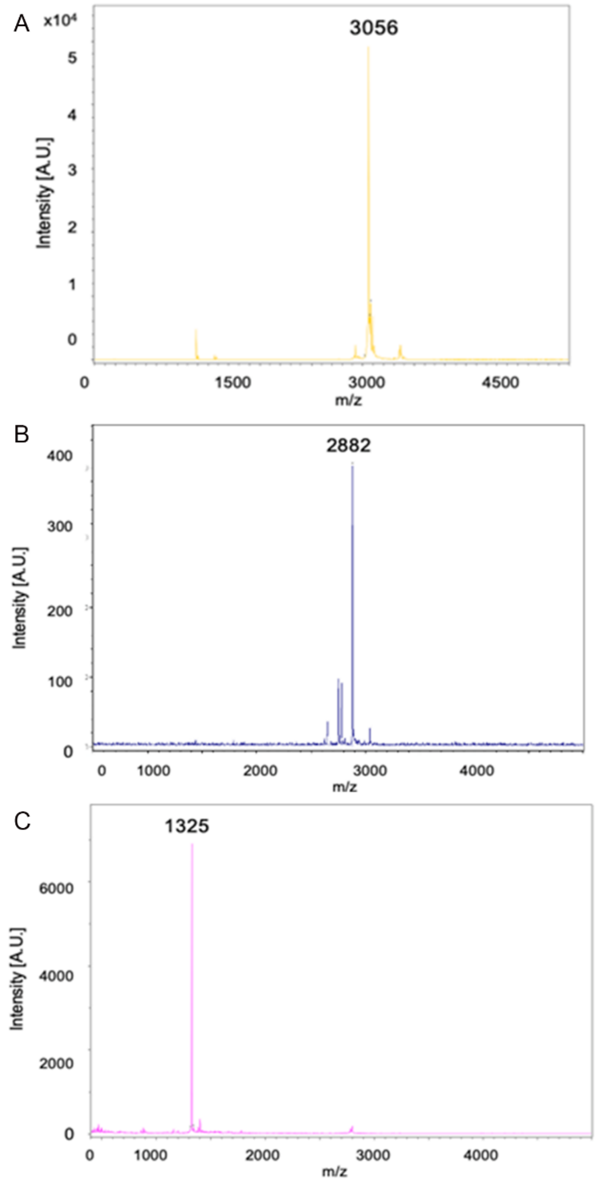

Supplementary Figure 3: MALDI characterization of P210 peptide (A, expected m/z: 3058), MSA peptide (B, expected m/z: 2882), and diC$_{16}$-cy7 (C, expected m/z: 1326) amphiphiles.

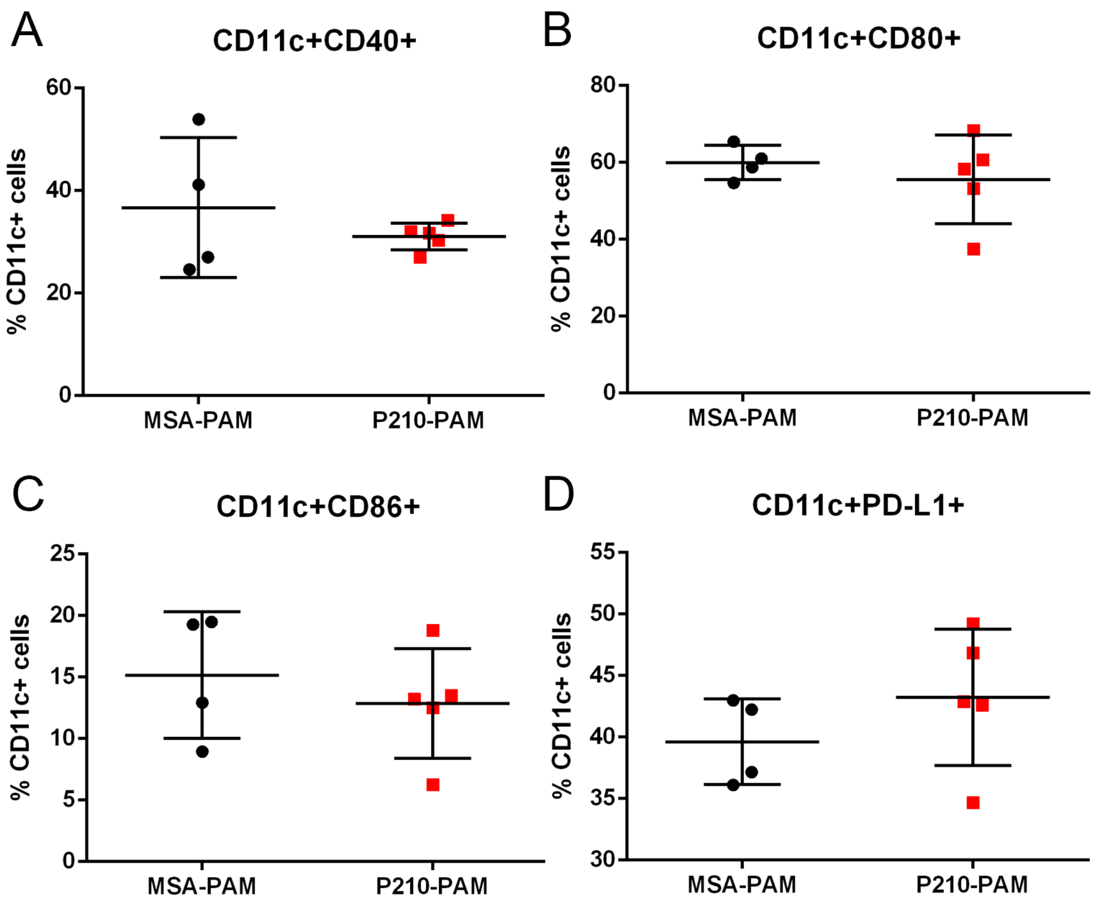

Supplementary Figure 4: Lack of effect on DC phenotype by P210-PAM immunization. (A) CD11c+CD40+, (B) CD11c+CD80+, (C) CD11c+CD86+, and (D) CD11c+PD-L1+ DCs were not significantly different between MSA-PAM (N=4) and P210-PAM (N=5) immunized *ApoE*$^{-/-}$ mice. Mice were sacrificed 1 week after second booster at 13 weeks of age.

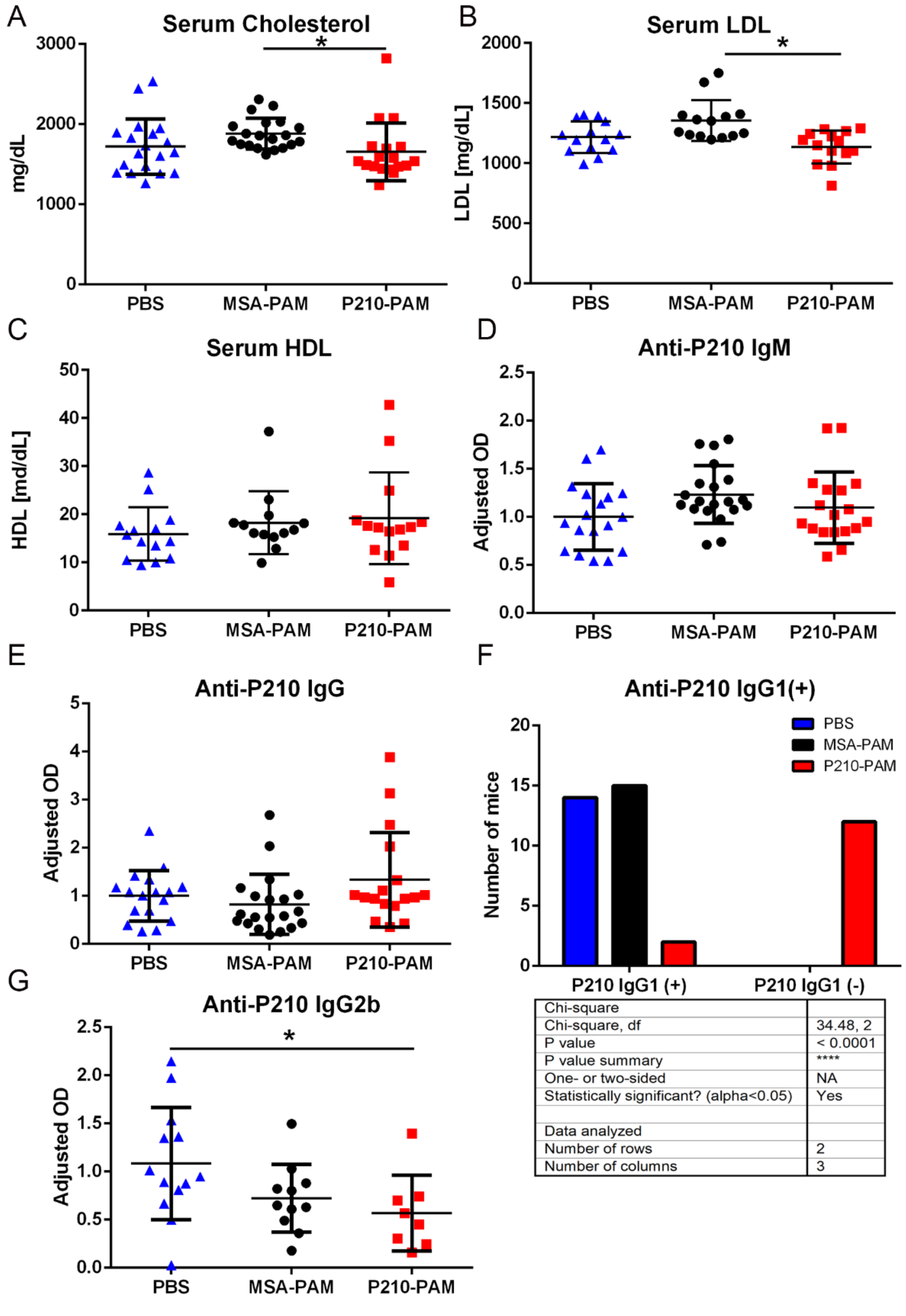

Chi-square	
Chi-square, df	34.48, 2
P value	< 0.0001
P value summary	****
One- or two-sided	NA
Statistically significant? (alpha<0.05)	Yes
Data analyzed	
Number of rows	2
Number of columns	3

Supplementary Figure 5: (A) Serum cholesterol, (B) serum LDL, (C) serum HDL, (D) anti-P210 IgM, and (E) anti-P210 IgG in immunized *ApoE*$^{-/-}$ mice. Anti-P210 isotypes (F) IgG1 and (G) IgG2b isotypes were further characterized. *P<0.05 Kruskal-Wallis followed by Dunn's multiple comparisons test except for (D); Chi-square for IgG1 to compare presence or absence of detectable anti-P210 IgG1; P<0.0001.

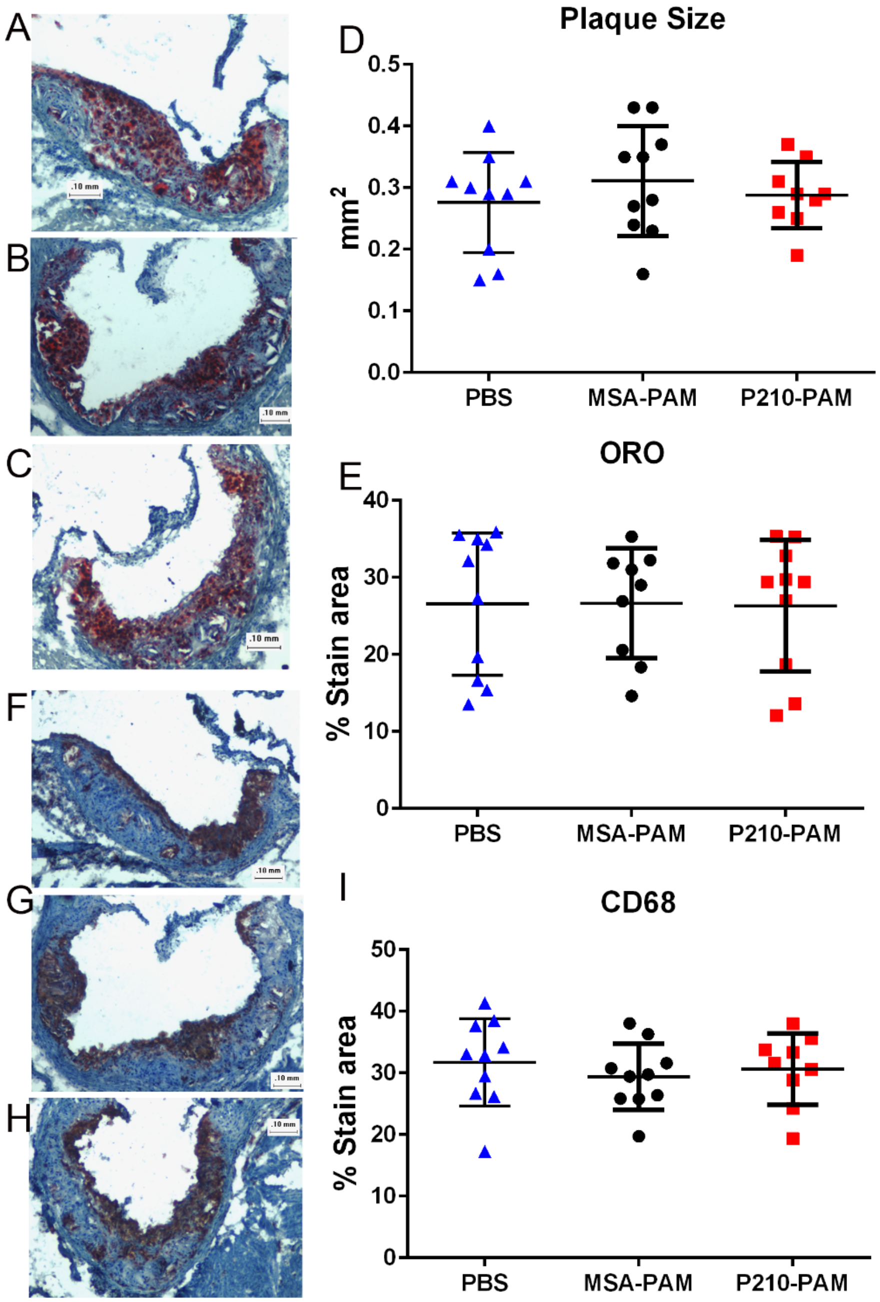

Supplementary Figure 6: Representative photos of Oil Red-O (ORO) stained aortic sinus plaques from (A) PBS, (B) MSA-PAM and (C) P210-PAM immunized *ApoE*$^{-/-}$ mice. (D) Plaque size and (E) ORO percent stained area measurements. Representative photos of CD68 stained aortic sinus plaques from (F) PBS, (G) MSA-PAM and (H) P210-PAM mice. (I) CD68 percent stained area measurements. Bar = 0.1 mm.

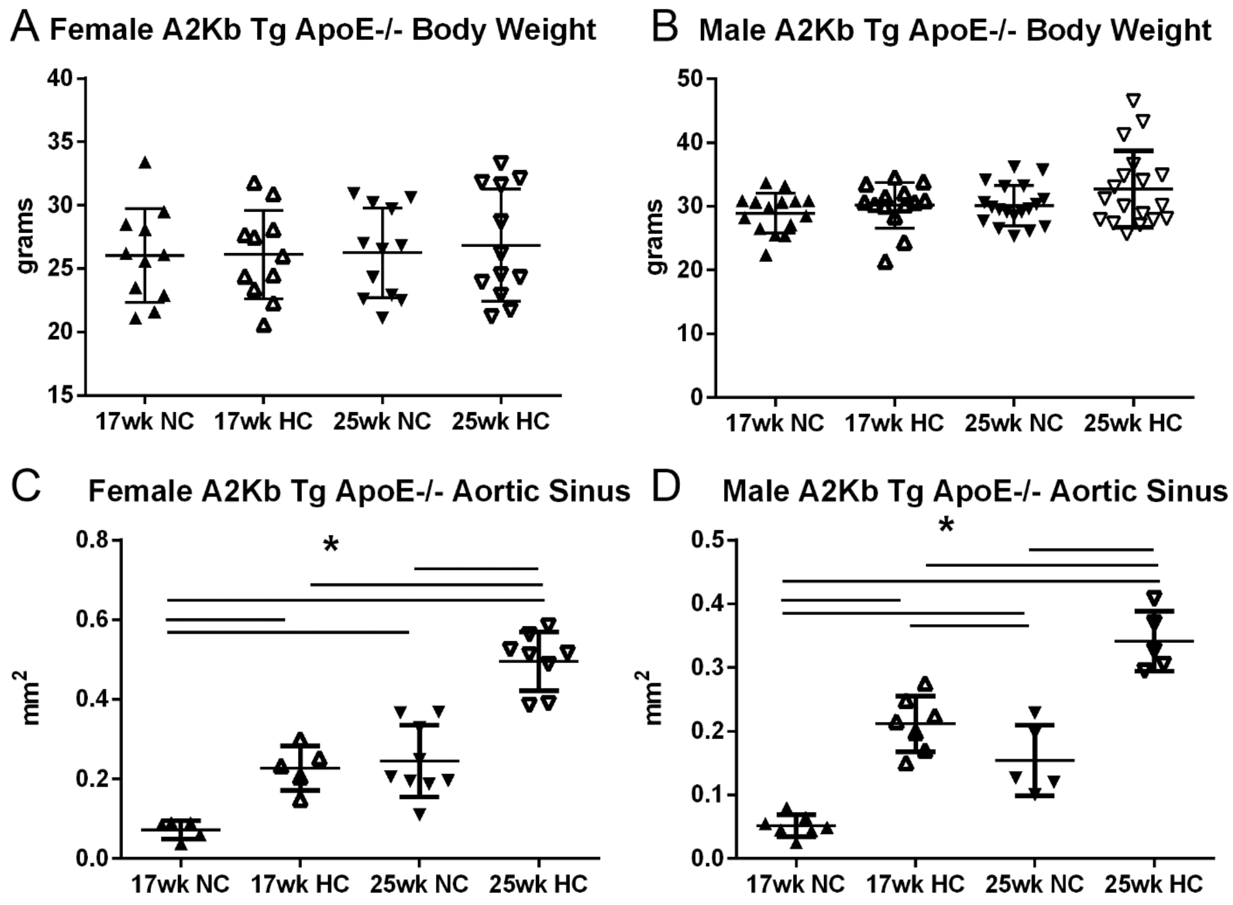

Supplementary Figure 7: (A) Body weight of female *A2Kb Tg ApoE*$^{-/-}$ and (B) male *A2Kb Tg ApoE*$^{-/-}$ mice at 17 weeks or 25 weeks of age fed with normal chow (NC) or high cholesterol diet (HC) for 8 or 16 weeks. (C) Aortic sinus plaque size in female *A2Kb Tg ApoE*$^{-/-}$ mice and (D) male *A2Kb Tg ApoE*$^{-/-}$ mice at 17 weeks or 25 weeks of age fed with normal chow (NC) or high cholesterol diet (HC) for 8 or 16 weeks. ANOVA with Holm-Sidak multiple comparisons test. *P<0.05

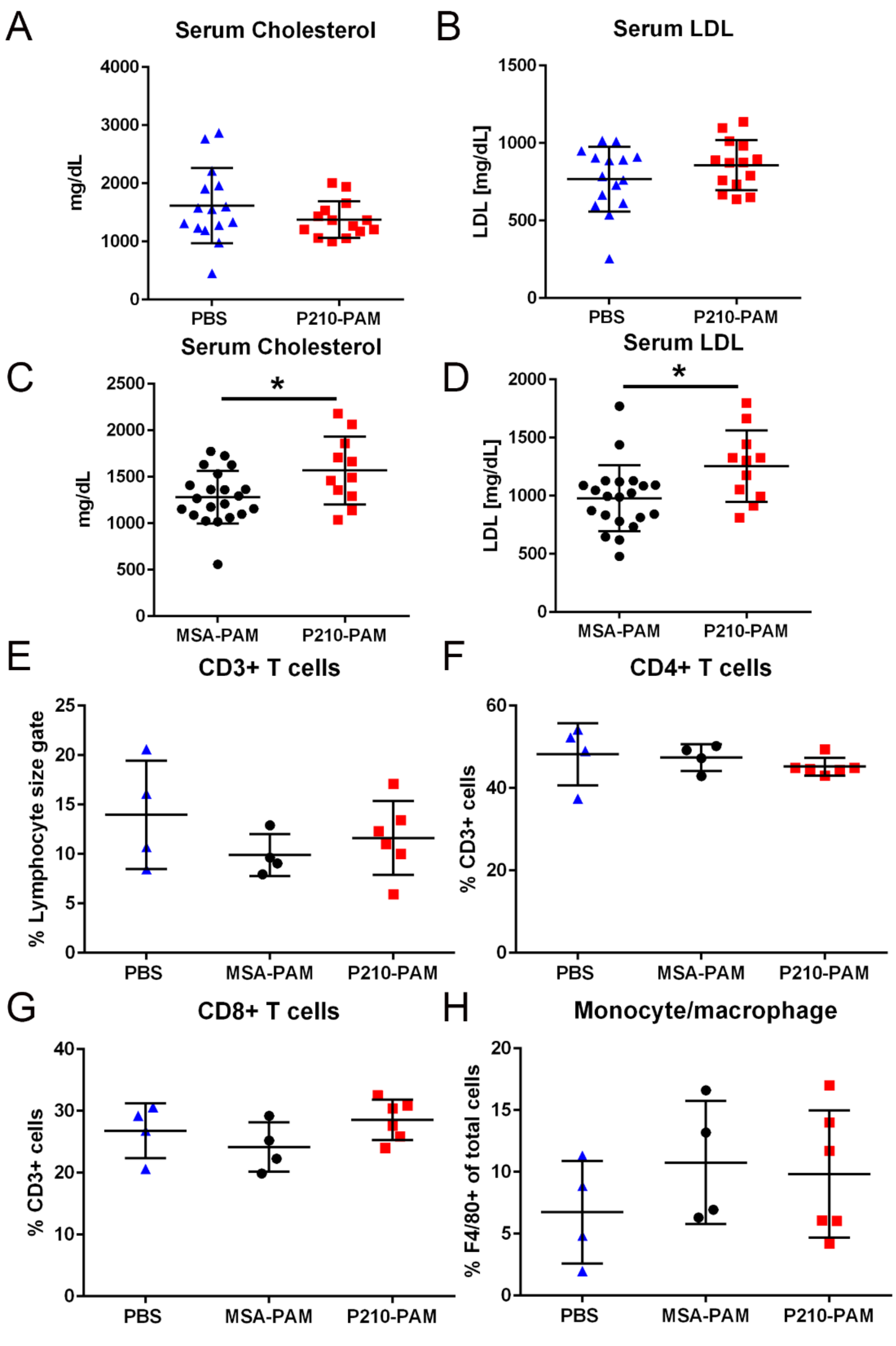

Supplementary Figure 8: (A & C) Serum total cholesterol and (B & D) serum LDL levels in immunized *A2Kb Tg ApoE*$^{-/-}$ mice. **P*<0.05. (E-H) Aortas from 25 weeks old immunized *A2Kb Tg ApoE*$^{-/-}$ mice of each group were subjected to enzymatic digestion and the recovered cells stained for (E) CD3, (F) CD4, (G) CD8, and (H) F4/80 for monocyte/macrophage.

Supplementary Table

Supplementary Table 1 **Antibodies used in AIM assay**

Marker	Fluorochrome	Clone	Manufacture
**CD4**	BUV395	SK3	BD Biosciences
**CD8a**	eFluor450	RPA-T8	Invitrogen
**CD25**	FITC	M-A251	BD Biosciences
**CD69**	PE	FN50	BD Biosciences
**CD134 (OX40)**	APC	ACT35	BD Biosciences
**CD137**	APC-eFluor 780	4B4 (4B4-1)	Invitrogen
**CD154 (CD40L)**	BV711	24-31	BioLegend
**CD14**	eFluor506	61D3	Invitrogen
**CD16**	eFluor506	eBioCB16	Invitrogen
**CD19**	eFluor506	HIB19	Invitrogen

Supplementary Table 2 Antibodies used for human effector memory T cells

	Fluorochrome	Clone	Manufacturer
**Viability**	Aqua Blue		Invitrogen
**CD3**	eF450	UCHT1	Invitrogen
**CD4**	BUV395	SK3	BD Biosciences
**CD8a**	APC	SK1	BioLegend
**CD45RA**	FITC	L48	BD Biosciences
**CD45RO**	APC-eF780	UCHL1	Invitrogen
**CD62L**	BV711	DREG-56	Invitrogen
**CD197**	PE	150503	BD Biosciences

Supplementary Table 3 Primers used in qPCR

Gene	Forward	Reverse
**GAPDH**	atcactgccacccagaagac	cacattgggggtaggaacac
**arginase 1**	ggcagaggtccagaagaatg	gccagagatgcttggaactg
**iNOS**	agtggtccaacctgcaggtc	ctgatgttgccattgttggt
**MCP-1**	cagccagatgcagttaacgc	gcctactcattgggatcatcttg
**IL-6**	ctgcaagagacttccatccag	agtggtatagacaggtctgttgg
**IL-10**	tttgaattccctgggtgagaa	acaggggagaaatcgatgaca
**IL-12**	cacgctacctcctctttttg	cagcagtgcaggaataatgtt
**IL-1β**	gggcctcaaaggaaagaatc	ttgcttgggatccacactct
**IL-1R1**	caggagaagtcgcaggaagt	tggaacagagccagtgtcag
**IL-17a**	tctctgatgctgttgctgct	cgtggaacggttgaggtagt

Supplementary Method

Amphiphile synthesis

Peptide amphiphiles were synthesized by conjugating peptides to the 1'-3'-dihexadecyl *N*-succinyl-L-glutamate (diC_{16}) hydrophobic tail (Joo J et al. Molecules. 2018;23:2786). DiC_{16} was synthesized by first mixing hexadecanol (22.4 g, 0.092 mol), L-glutamic acid (6.8 g, 0.047 mol), and para-toluenesulfonic acid (10.5 g, 0.051 mol) to yield 1'-3'-dihexadecyl L-glutamate, which was then purified through Buchner funnel filtration through acetone and identified through ^{1}H-NMR, as shown in Supplementary Figure 2A. This was then mixed with succinic anhydride in 1:1 tetrahydrofuran:chloroform to yield 1'-3'-dihexadecyl *N*-succinyl-L-glutamate (diC_{16}). The crude diC_{16} was then crystallized overnight at 4 ^{o}C, purified through Buchner funnel filtration through diethyl ether, and identified via ^{1}H-NMR, as shown in Supplementary Figure 2B.

One mmol of P210 or mouse serum albumin (MSA; QTALAELVKHKPKATAEQLK) peptides were synthesized on an automated peptide synthesizer (PS3, Protein Technologies, Tucson, AZ, USA) with Fmoc-mediated solid phase peptide synthesis. Then peptides were conjugated to 1 mmol diC_{16} overnight through a peptide bond using *N,N*-diisopropylethylamine (1.25 mmol) and *O*-benzotriazole-*N,N,N',N'*-tetramethyl-uronium-hexafluoro-phosphate (1.125 mmol). Peptide amphiphiles were then cleaved from the solid phase resin by shaking in a 95:2.5:2.5 % volume trifluoroacetic acid:triisopropylsilane:water solution for 2 hours, precipitated in ice-cold diethyl ether, and lyophilized. Peptide amphiphiles (PA) were purified using reverse-phase, high-pressure liquid chromatography (RP-HPLC, Prominence, Shimadzu, Columbia, MD, USA) on a Luna C4 column (Phenomenex, Torrance, CA, USA) at 55 ^{o}C with 0.1% formic acid in water and

acetonitrile mixtures as mobile phases. The purity of eluted peptide amphiphiles was characterized using matrix-assisted laser desorption/ionization time-of-flight mass spectroscopy (MALDI-TOF-MS, Bruker, MA, USA). As shown in Supplementary Figure 3A, the expected mass peak for the P210 PA is 3058 g/mol, and as shown in Supplementary Figure 3B, the expected mass peak for the MSA PA is 2882 g/mol. Fluorescently labeled diC_{16}-cy7 amphiphiles were synthesized by reacting diC_{16} with cy7-amine, 1-ethyl-3-(3-dimethylaminopropyl)-carbodiimide (EDC), *N*-hydroxysuccinimide (NHS), and triethanolamine (TEA) in dimethyl sulfoxide (DMSO) at a 1:1.5:4:1:1 ratio diC_{16}:cy7-amine:EDC:NHS:TEA. The EDC, NHS, and TEA were divided into five aliquots, with the first four aliquots added sequentially 2 h after the previous aliquot, while the fifth aliquot was added 12 h after the fourth aliquot. Afterwards, the reaction was stirred for an additional 24 h before purification through RP-HPLC. As shown in Supplementary Figure 3C , the expected mass peak for the diC_{16}-cy7 is 1326 g/mol.

Micelle assembly

Micelles were prepared through thin-film hydration as previously reported (Joo J et al. Molecules. 2018;23:2786). Briefly, peptide amphiphiles were dissolved and sonicated in methanol, before evaporation under a nitrogen stream into thin films. Films were hydrated in water or PBS, sonicated and heated to 80 ^{0}C for 30 minutes before cooling to room temperature. Fluorescently labeled P210 or MSA PAMS were synthesized by mixing P210 or MSA PAs with diC_{16}-cy7 at a 90:10 molar ratio.

Micelle characterization

The shape and morphology of micelles were characterized through transmission electron microscopy (TEM). Seven µL of 100 µM P210 PAMs was placed onto 400 mesh carbon

grids (Ted Pella, Redding, CA, USA) for 5 minutes, before excess liquid was wicked, and the grids were washed with water. The grids were then stained 2% uranyl acetate, washed again with water, and dried before imaging on a JEOL JEM 2100-F TEM (JEOL, Tokyo, Japan). Micelle size, polydispersity, and zeta potential were characterized using a Dynapro Nanostar system (Wyatt, Santa Barbara, CA, USA). One hundred µM of micelles were suspended in water and placed in a quartz cuvette with a platinum dip probe (n = 3) for size, polydispersity, and zeta potential analysis.

Preparation of chimeric A2Kb gene DNA fragments for fertilized eggs microinjection

A 3867bp full-length chimeric A2Kb gene containing sequence coding the leader sequence, α1 and α2 domains of HLA-A*02:01 and α3, transmembrane and cytoplasmic domains of the mouse MHC I H-2Kb gene (intron 3 to intron 8) was cloned by PCR using 35 cycles of 94°C for 50s, 56°C for 50s, and 68°C for 4min, with the genomic DNA from A2Kb transgenic CB6F1-Tg(HLA-A*02:01/H2-Kb)A*02:01 mouse as template (Figure A). The following primers were used: F 5'-ATCAAGCTTACTCTCTGGCACCAAAC-3', R 5'-TAAGGATCCCTAGTTGAGTCTCTGA-3'.

A2Kb PCR products purified with Monarch® DNA Gel Extraction Kit (New England Biolabs, Cat # T1020S) were ligated with pCR-XL-TOPO T vectors (Figure B) and transformed into competent *E. Coli* cells following the kit's protocol (Thermo Fisher, Cat # K4750-10).

Recombinant plasmid clone with the right chimeric A2Kb sequence (sequenced by Laragen, Culver City, CA 90232) was amplified and purified by using Qiagen Plasmid Midi Kit (Cat #12143), following the protocol. Chimeric A2Kb fragments for microinjection were prepared by digesting 10-20ug of the purified recombinant plasmids with restriction enzymes

Hind III, BamH I and Hinc II (New England Biolabs, Cat # R0104S, R0136S, R0103S), at 37^0C for 4hrs, followed by purification of the resulted A2Kb DNA fragments.

Figure A

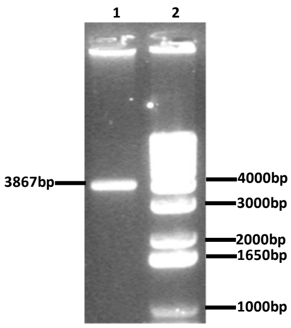

Figure B

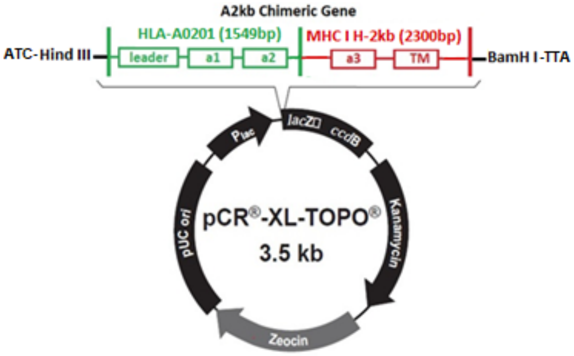

Figure C

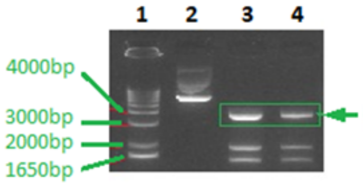

Figure A: PCR products of A2Kb chimeric gene (Lane 1: A2Kb product; Lane 2: 1kb plus DNA ladder)

Figure B: Cloning of A2Kb chimeric gene into pCR-XL-TOPO T vector

Figure C: A2Kb fragments obtained by digesting recombinant plasmids with Hind III, BamH I and Hinc II (Lane1: 1kb plus DNA ladder; Lane 2: Products of digesting recombinants with Hinc II; Lane 3 & 4: Products of digesting recombinants with Hind III, BamH I and Hinc II; green arrow indicate the fragments purified and used for fertilized egg microinjection).

Generation of A2Kb transgenic founder by fertilized eggs microinjection and selection of A2Kb transgenic offspring for experiments

Purified A2Kb fragments (~3.9-kb, Figure C) obtained as described were then microinjected into *ApoE*$^{-/-}$ fertilized eggs by Rodent Genetics Core at Cedars-Sinai Medical Center. Germline-transmitted A2Kb chimeras obtained were screened by PCRs detecting 148bp, 309bp, 252bp, 195bp fragments coding part of leading peptide, α1, α2 domains of HLA-A*02:01 (Figure D & E) using toe genomic DNA (prepared by QuickExtract^{TM} DNA Extraction Solution, Epicentre, Cat#QE09050) as template. Primers used for PCR are listed as following: HLA A*02:01 leader (148bp): F 5'- ACTCAGATTCTCCCCAGACGC-3' and R 5'-CCGTTGCTTCTCCCCACAGAG-3'; HLA A*0201 a1 (309bp): F 5'-TGTGGGGAGAAGCAACGGG and R 5'-GAGTGGGCCTTCACTTTCCG; HLA A*0201 a2 (195bp): F 5'-GTTCTCACACCGTCCAGAGGAT-3' and R 5'-ACTGCTCCGCCACATGGGCCGC-3'; HLA A*0201 a2 (252bp): F 5'- TACCACCAGTACGCCTACGA-3' and R 5'-ATCTACAGGCGATCAGGGAG-3'.

PCR results revealed one male chimera's genomic DNA might carry the A2Kb chimeric gene. A2Kb protein expression on the surface of peripheral blood mononuclear cells (PBMCs) in this mouse was further verified by flow cytometric analysis of cells stained with anti-human HLA-A2 (FITC, Clone: BB7.2, BD Bioscience, Cat#551285) and anti-mouse MHC-I H2Kb (PE, Clone AF6-88.5, BD Biosciences, Cat# 553570). PBMCs from Taconic A2Kb Tg mice or *ApoE*$^{-/-}$ mice were used as positive and negative control respectively (Figure F).

The identified A2Kb transgenic *ApoE*$^{-/-}$ male mouse then crossbred with female *ApoE*$^{-/-}$mice, the A2Kb transgenic offspring used for further breeding and experiments were selected by flow cytometric analyzing the expression of chimeric A2Kb protein on surface of PBMCs. RT-PCR

detecting a 1092bp fragment of A2Kb mRNA (1113bp of full-length) expression in splenic total RNAs of such A2Kb (+) offspring was further used to verify integration of the full-length A2Kb gene into the mouse genome (Figure G), following a PCR program of 35 cycles of 94^{0}C, 1min; 62^{0}C, 45sec; 72^{0}C, 1.5min. Primers used for RT-PCR are F 5'-AACCCTCGTCCTGCTACTCT-3' and R 5'-CACGCTAGAGAATGAGGGTCA-3'.

Figure D

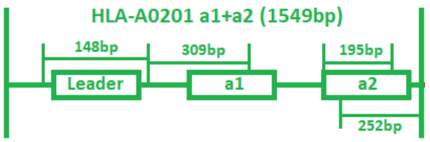

Figure F

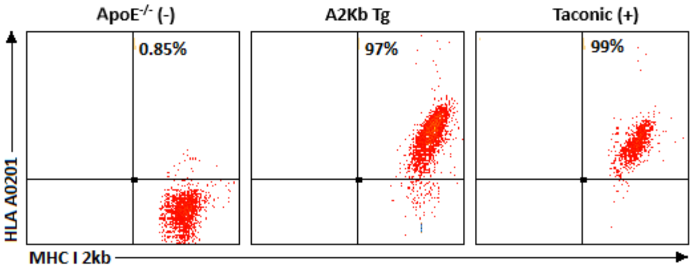

Figure E

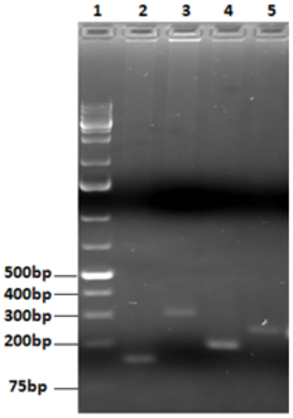

Figure G

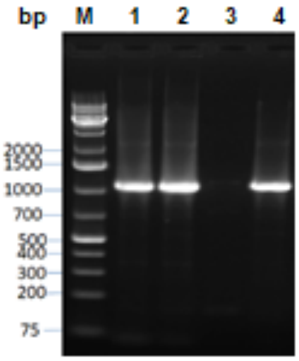

Figure D: HLA A*0201 fragments used for PCRs screening A2Kb transgenic *ApoE*$^{-/-}$ mice

Figure E: Chimeras carrying A2Kb gene were screened by PCR detecting HLA A*02:01 fragments (Lane1: 100bp ladder; Lane 2: 148bp; Lane 3: 309bp; Lane 4: 252bp; Lane 5: 195bp).

Figure F: Flow cytometric analysis of A2Kb expression on PBMCs of A2Kb transgenic *ApoE*$^{-/-}$ mice.

Figure G: RT-PCR detecting a 1092bp A2Kb mRNA fragment show full-length of A2Kb gene been integrated into the mice genome and efficiently transcribed (Lane 1, 2 & 4: A2Kb(+) offspring; Lane 3: A2Kb(-) offspring).
